# Supplementary material for: Metabolic enzyme cost explains variable trade-offs between microbial growth rate and yield
Source: PLoS Comput Biol. 2018 Feb 16;14(2):e1006010. doi: 10.1371/journal.pcbi.1006010 (PMC5847312; doi:10.1371/journal.pcbi.1006010)
Supplement: S1 Text — (PDF) [file pcbi.1006010.s001.pdf]

Metabolic enzyme cost explains variable trade-offs between microbial growth rate and yield: Supplementary information

Meike T. Wortel[1,2,†], Elad Noor[3,†], Michael Ferris[4], Frank J. Bruggeman[2], and Wolfram Liebermeister[5,6,*]

[1]Centre for Ecological and Evolutionary Synthesis (CEES), Department of Biosciences, University of Oslo, Oslo, Norway
[2]Systems Bioinformatics Section, Amsterdam Institute for Molecules, Medicines and Systems (AIMMS), Vrije Universiteit, Amsterdam, The Netherlands
[3]Institute of Molecular Systems Biology, Eidgenössische Technische Hochschule, Zürich, Switzerland
[4]Computer Sciences Department and Wisconsin Institute for Discovery, University of Wisconsin, Madison, USA
[5]INRA, UR1404, MaIAGE, Université Paris-Saclay, Jouy-en-Josas, France
[6]Institute of Biochemistry, Charité – Universitätsmedizin Berlin, Berlin, Germany
[†]M.W. and E.N. contributed equally to this work
[*]wolfram.liebermeister@inra.fr

Contents

1 Methods descriptions **3**

1.1 Parameter balancing . . . 3
1.2 Enzyme cost minimization implemented in the GAMS modeling system . . . 3
1.3 Converting enzyme investments into cell growth rates . . . 4
1.4 EFCM provides advantages over common kinetic or constraint-based modeling methods . . . 7
1.5 A kinetic description of enzyme capacity utilization . . . 10
1.6 Extending EFCM to larger models and multiple enzyme cost functions . . . 10

2 Model description **11**

2.1 Network structure . . . 11
2.2 Kinetic equations . . . 12
2.3 Choice of consistent model parameters by parameter balancing . . . 14
2.4 Calculation of specific growth rate and yield . . . 14
2.5 Absolute values for enzyme concentrations . . . 15
2.6 Choice of standard external conditions . . . 15
2.7 Details on the elementary flux modes . . . 16

3 Model results **19**

3.1 Growth rates achieved by elementary flux modes . . . 19
3.2 Effect of individual enzyme parameters on cell growth . . . 19
3.3 Monod curves and optimal EFMs under high-glucose or low-glucose conditions . . . 21
3.4 Growth of strains deficient in EMP glycolysis, ED glycolysis, or respiration . . . 23
3.5 Epistatic effects between enzyme knockouts . . . 26

4 Mathematical derivations **33**

4.1 Computing a stationary flux distribution in a metabolic network from measured fluxes . . . 33
4.2 Global cost sensitivities can be approximated by local cost sensitivities . . . 33
4.3 Enzyme cost sensitivities of kinetic constants . . . 34
4.4 Growth-rate sensitivities of kinetic constants . . . 36
4.5 Increasing a k_{cat} value cannot increase the enzyme demand . . . 37
4.6 Approximation formula for Monod curves and higher-dimensional Monod functions . . . 37

5 Model details and elementary flux mode statistics **40**

5.1 Tables with model details . . . 40
5.2 Sampling of flux modes near the Pareto front . . . 44
5.3 Selected elementary flux modes . . . 44

6 List of supplementary data files **51**

1 Methods descriptions

This section provides some details about different parts of our modeling approach: parameter balancing for obtaining kinetic parameters; a fast implementation of enzyme cost minimization; the conversion between enzyme-specific biomass production into cell growth rates; and a comparison of EFCM to traditional kinetic and constraint-based modeling approaches.

1.1 Parameter balancing

Parameter balancing [1] is a method for translating incomplete and possibly inconsistent sets of kinetic constants into a complete, consistent set of model parameters. It works as follows. We collect all relevant quantities that appear in the data or in the model (e.g. $k_{\rm cat}$, $K_{\rm eq}$, or $k_{\rm M}$ values) and merge them into a vector $\mathbf{y}$. The model parameters must satisfy Wegscheider conditions and Haldane relationships, which define linear equality constraints between their logarithmic values. To satisfy these constraints, we write all these quantities as linear combinations of independent parameters ($\ln k^{\rm V}$, $\ln k_{\rm M}$, and μ° values), with the definition $k^{\rm V} = \sqrt{k_{\rm cat}^{+}\, k_{\rm cat}^{-}}$. The independent parameters, which we collect in a vector $\mathbf{x}$, can now be varied without violating any constraints. The linear dependence between complete and independent parameter sets can be written as $\mathbf{y} = \mathbf{R}\,\mathbf{x}$, with a matrix $\mathbf{R}$ derived from the model structure. Using this equation as a linear regression model, we can convert an experimentally known vector $\mathbf{y}^{\rm data}$ (which may be incomplete) into a best estimate of the underlying vector $\mathbf{x}$. Using the estimate $\mathbf{x}$, and multiplying again by $\mathbf{R}$, we obtain a completed, consistent version of $\mathbf{y}$. Since the regression problem is usually underdetermined, we employ Bayesian priors to obtain plausible estimates even from sparse data. Accordingly, the result is not simply a point estimate of $\mathbf{y}$, but a multivariate Gaussian posterior distribution for the possible parameter vectors $\mathbf{y}$. A best estimate is obtained from the center of the distribution (or its maximum point, if further constraints are applied); from the covariance matrix, we obtain uncertainties of individual model parameters as, well as the correlations between them. For details and an applicable online tool, see www.parameterbalancing.net.

1.2 Enzyme cost minimization implemented in the GAMS modeling system

Finding the minimal enzyme cost for an individual EFM is a convex problem and can therefore be solved with local optimization methods and in polynomial time. This allows us to use a powerful solver that optimizes a single enzyme profile in a few seconds. We have implemented enzyme cost minimization within the General Algebraic Modeling System (GAMS) modeling system [2], accessible through the NEOS server (`https://neos-server.org/neos/`), which provides a convenient way to write down the optimization problem and uses automatic differentiation techniques to exactly evaluate derivatives, e.g. of the functions defining the constraints and objective function of the model. This is important since it allows nonlinear optimization solvers to efficiently use this information for improved solution speed and accuracy. To define an optimization problem, the user provides the metabolic network (in the form of reaction stoichiometries), kinetic constants, fluxes, and possibly enzyme costs. The data can be provided as a single csv file or a collection of csv files, and the user can select files defining the model and its parameters, as well as one of the optimization solvers linked to the GAMS system. As a default setting, general rate equations as in this paper are used (modular rate laws [3], possibly with simple allosteric regulation). However, the implementation also allows for custom rate equations, such as the biomass equation in this paper (a case study with explanations can be found on `http://www.neos-guide.org/content/enzyme-cost-minimization`). Additional arguments allow for defining bounds on subsets of the decision variables, changing the solver, and setting options for the solver. We used metabolite bounds of e^{-20} to $e^{5.5}$ and enzyme bounds of 0 to e^{20} in our simulations.

Quantity	Symbol	Physical unit
Metabolite level (metabolite i)	c_i	mM
Metabolite flux (reaction l)	v_l	mM s^{-1}
Enzyme concentration (enzyme l)	E_l	mM
Biomass production	v_{BM}	mg l^{-1} h^{-1}
Total enzyme cost	E_{met}	mg l^{-1}
Enzyme doubling time	τ_{met}	h
Enzyme specific biomass production	r_{BM}	h^{-1}
Cellular protein mass	P_{tot}	mg l^{-1}
Biomass concentration	c_{BM}	mg l^{-1}
Protein/biomass mass fraction	f_{prot}	unitless
Enzyme/protein mass fraction	f_{ccm}	unitless
Doubling time	T	h
Cell growth rate	μ	h^{-1}

Table S1: Mathematical symbols and physical units used in the formulae.

Although ECM is applied here to EFMs only, it can be used to compute the enzyme cost of any given flux mode. The calculations for this article were executed on a shared server: a Dell PowerEdge R430 server with the following configuration: CPU - 2x Intel Xeon E5-2698 @ 2.3GHz (32 cores total), HT Enabled, Memory - 192GB RAM, Disk - 4x 300G SAS drives setup in RAID5, Network - 1Gb/s Ethernet.

1.3 Converting enzyme investments into cell growth rates

Being able to compute enzyme-specific biomass production rates, we next translate these rates into cell growth rates. The growth rate of a cell is given by $\mu = v_{\mathrm{BM}}/c_{\mathrm{BM}}$, where c_{BM} is the biomass concentration (i.e. the amount of biomass per cell volume) and v_{BM} is the rate of biomass production (i.e. the amount of biomass produced per cell volume and per unit time). The cell's growth rate can be approximated based on the enzyme cost of biomass production, where higher enzyme-specific biomass production rates entail higher growth rates. Therefore, an assessment of enzyme-specific biomass production rates (in optimization, or in the rate/yield scatter plots) is equivalent to an assessment of growth rates. Here we derive two conversion formulae: a linear formula, which assumes a constant amount of metabolic enzyme within the biomass, and a nonlinear one, which takes into account the growth-rate-dependent investment in ribosomes. As shown in Figure S1, predicted growth rates yield a overall picture similar to the direct assessment of enzyme-specific biomass production.

Linear growth rate formula based on a fixed proteomic enzyme fraction

To estimate $\mu = v_{\mathrm{BM}}/c_{\mathrm{BM}}$ from the enzyme-specific biomass production $r_{\mathrm{BM}} = v_{\mathrm{BM}}/E_{\mathrm{met}}$, we need to know the ratio $E_{\mathrm{met}}/c_{\mathrm{BM}}$, i.e. the fraction of biomass formed by metabolic enzymes. Empirically, metabolic enzymes (or more precisely: the metabolic enzymes considered in our model) occupy about one eighth of the biomass (in mass units). The mass fraction of protein within biomass, $f_{\mathrm{prot}} = P_{\mathrm{tot}}/c_{\mathrm{BM}} \approx 0.5$ (BioNumber 101955 [4]), is relatively constant across cell types. The mass fraction of metabolic enzyme within the proteome, in *E. coli*, varies around $f_{\mathrm{ccm}} = E_{\mathrm{met}}/P_{\mathrm{tot}} \approx 25\%$ (from proteomics data [5]). If these numbers were constant, the growth rate

$$\mu = \frac{v_{\mathrm{BM}}}{c_{\mathrm{BM}}} = \underbrace{\frac{v_{\mathrm{BM}}}{E_{\mathrm{met}}}}_{r_{\mathrm{BM}}} \underbrace{\frac{E_{\mathrm{met}}}{P_{\mathrm{tot}}}}_{f_{\mathrm{ccm}}} \underbrace{\frac{P_{\mathrm{tot}}}{c_{\mathrm{BM}}}}_{f_{\mathrm{prot}}} \tag{1}$$

would be proportional to the enzyme-specific biomass production r_{BM}, with a prefactor of $f_{\mathrm{ccm}} \cdot f_{\mathrm{prot}} \approx 0.125$:

$$\mu \approx 0.125\, r_{\mathrm{BM}}. \tag{2}$$

Nonlinear growth rate formula based on growth-dependent enzyme fraction

In reality, the amount of metabolic enzyme within the proteome is subject to change. As shown by experiments and as explained by resource allocation models [6], the fraction of metabolic enzymes decreases with the growth rate, at least if the growth rate changes are caused by varying metabolic efficiency (e.g. due to different carbon sources). We can account for this changing fraction by using a modified formula. In experiments where cell growth is controlled by nutrient quality or by dilution in chemostats, we can assume a linearly decreasing fraction [6]

$$\frac{E_{\mathrm{met}}}{P_{\mathrm{tot}}} = a - b\,\mu \tag{3}$$

with positive coefficients a and b. These coefficients can be estimated from proteomics data [5]: the protein fraction devoted to core carbon metabolism decreases from $\approx$ 25% during slow growth (μ = 0.11/h) to $\approx$ 18% during faster growth (μ = 0.48/h), leading to estimates $a \approx 27\%$ and $b \approx 20\%$ h. Inserting (3) into (4.6) and solving for μ, we obtain the formula

$$\mu = \frac{a\, f_{\mathrm{prot}}\, r_{\mathrm{BM}}}{1 + b\, f_{\mathrm{prot}}\, r_{\mathrm{BM}}}. \tag{4}$$

By inserting the numerical values, we obtain

$$\mu \;=\; \frac{0.27 \cdot 0.5 \cdot r_{\mathrm{BM}}}{1 + 0.2[\mathrm{h}] \cdot 0.5\, r_{\mathrm{BM}}} = \frac{0.135\, r_{\mathrm{BM}}}{1 + 0.10[\mathrm{h}] \cdot r_{\mathrm{BM}}}. \tag{5}$$

In our approximation formulae (2) and (5), μ increases with r_{BM}. This is why we claim that maximizing the growth rate μ is equivalent to maximizing the ratio $r_{\mathrm{BM}} = v_{\mathrm{BM}}/E_{\mathrm{met}}$, or minimizing E_{met} at given v_{BM}. As shown in Figure S1, the nonlinearity introduced by the second formulae has no effects on the qualitative rate/yield trade-offs. The derivation of Eqs (4) and (4.6) is illustrated in Figure S1(c). Finally, we can rewrite formulae (2)-(5) in terms of doubling time. For that, we define the *metabolic enzyme doubling time* as

$$\tau_{\mathrm{met}} \equiv \frac{\ln(2) E_{\mathrm{met}}}{v_{\mathrm{BM}}} = \frac{\ln(2)}{r_{\mathrm{BM}}}, \tag{6}$$

and the cell doubling time (in hours) will thus be

$$T = \frac{\ln(2)}{\mu} = \frac{\tau_{\mathrm{met}}}{f_{\mathrm{prot}}\, a} + \frac{\ln(2) \cdot b}{a} = 7.4\, \tau_{\mathrm{met}} + 0.51[\mathrm{h}]. \tag{7}$$

Nonlinear growth rate formula derived from an enzyme-ribosome trade-off

In the previous section, we derived the nonlinear efficiency/growth relationship from an empirical observation: the fraction of metabolic enzymes within the proteome decreases linearly with the growth rate. Here we show an alternative derivation that directly refers to the resource allocation models by Scott *al.* [6]. To derive the nonlinear relationship (4) between enzyme-specific biomass production and cell growth rate, we assumed that the fraction of metabolic enzymes within the proteome decreases with the growth rate, and

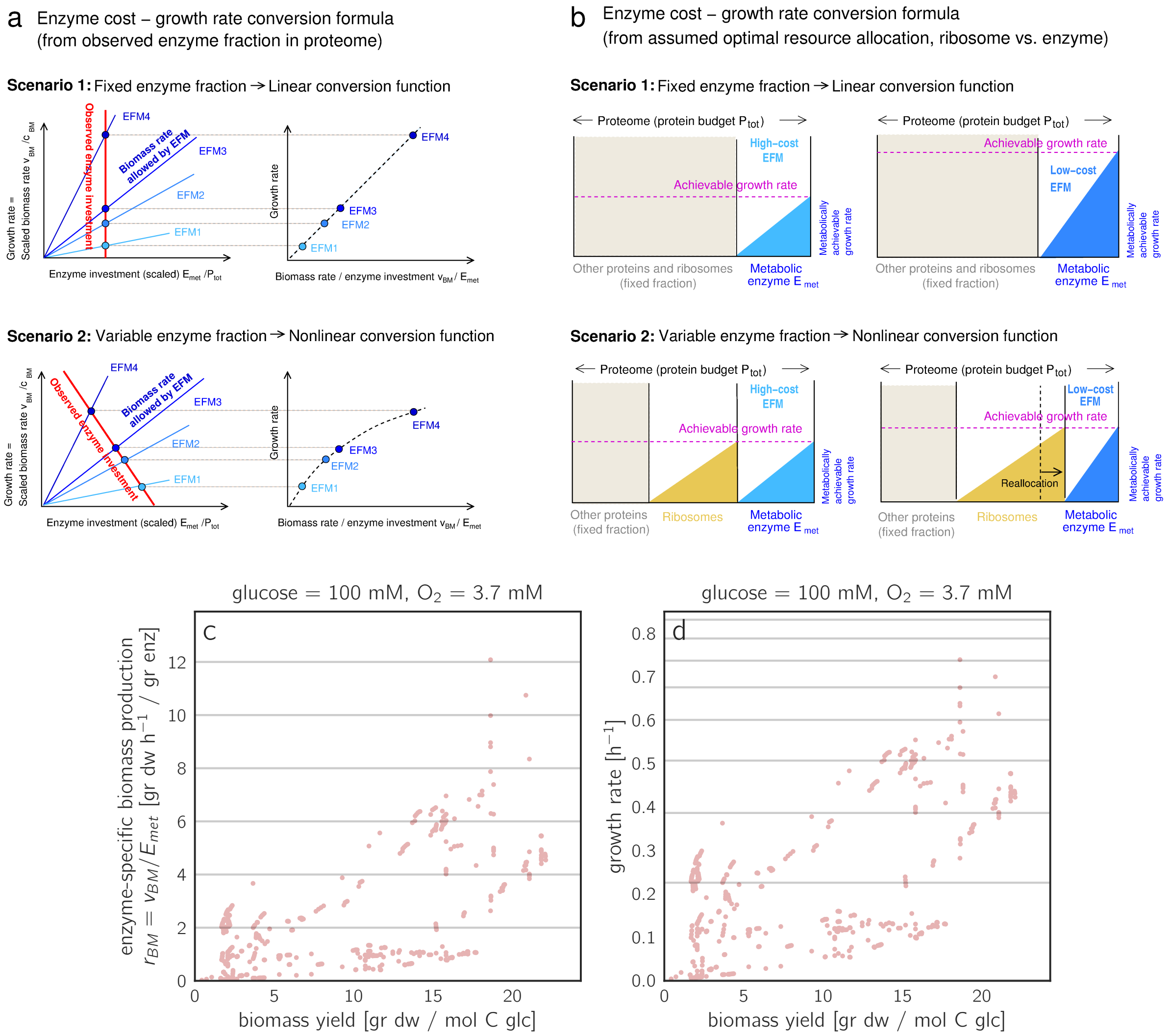

Figure S1: **Biomass production rates and growth rates.** (a) Cost/growth conversion formulae, derived by comparing the biomass production rate allowed by an EFM (blue line) to the empirically observed metabolic enzyme investment $E_{\mathrm{met}}/P_{\mathrm{tot}}$ at a given growth rate μ (red line). More precisely, blue lines show the scaled biomass rate $v_{\mathrm{BM}}/c_{\mathrm{BM}}$ as a function of scaled enzyme investment $E_{\mathrm{met}}/P_{\mathrm{tot}}$. Top left: We assume that the observed enzyme investment is constant (vertical red line). Since growth rate μ and scaled biomass production rate $v_{\mathrm{BM}}/c_{\mathrm{BM}}$ are identical (y-axis), we can match available enzyme budget (red line) and enzyme investment (blue line) for each EFM by taking the intersection point. Top right: For each EFM, we plot the achievable growth rate (y-axis coordinate of intersection point in left plot) against the biomass production rate per enzyme investment (slope of blue EFM line), scaled by the constant factor $P_{\mathrm{tot}}/c_{\mathrm{BM}}$ (assuming that P_{tot} is a fixed fraction of c_{BM}). The dots for all EFMs fall on a straight line, our linear conversion function Eq. (4.6). Bottom: Alternatively, we assume that the observed enzyme investment decreases linearly with the growth rate (diagonal red line, schematic drawing). Applying the same procedure, we obtain the nonlinear conversion function Eq. (4). (b) Alternative derivation of the conversion functions, assuming an optimal partitioning of protein resources into ribosomes and metabolic enzymes. Top: We assume that metabolic enzymes occupy a fixed fraction of the proteome. Each EFM defines a biomass rate per enzyme investment (slope of blue triangle, called a_x in Eq. (8); compare slopes in (a)). An EFM with a higher slope (drawing on the right) can support a proportionally larger growth rate, giving rise to our linear conversion function Eq. (4.6). Bottom: Now we assume a variable partitioning between ribosomes and metabolic enzymes, and that the growth rate is limited both by ribosomal and metabolic activity. The ribosomal efficiency (slope of brown traingle) is called a_Y in Eq. (8). Again, an EFM with a higher slope (right) supports a higher growth rate, but the effect is now less pronounced because of the reallocation of protein resources (arrow). This scenario leads, again, to the same nonlinear conversion function Eq. (4) as in (a). (c) Predicted enzyme-specific biomass production *versus* biomass yield computed for all EFMs. (d) Predicted cell growth rate *vs.* biomass yield. The nonlinear scaling (i.e. using equation (5)) of the growth rate has only minor effects on the Pareto plot. The horizontal grey lines are guides for the eye. On the left, the lines are evenly spaced, every 2 [gr dw h^{-1} / gr enz]. On the right, the same lines are converted to growth rates (in [h^{-1}]) and do not appear evenly spaced since the conversion is nonlinear (see Equation 4).

we described this dependence by a simple linear function (with offset) which we extracted from proteome data. This argument agrees with the resource allocation model by Scott *al.* [6], in which the proteomic fraction of metabolic enzymes is given by a constant baseline amount plus a variable amount proportional to the growth rate. To clarify this, we derive our formula again using the terminology of resource allocation models (see Figure S1(d)). We assume that the proteome can be split into three mass fractions: a constant fraction (about half of the proteome), a variable fraction x (consisting of metabolic enzymes), and a variable fraction y (consisting of ribosomal proteins). We further assume that each of the fractions x and y is proportional to the cell growth rate μ with different coefficients, i.e. $\mu = a_x\, x = a_y\, y$. Here a_x is given by (or proportional to) the enzyme-specific biomass production rate (which we can compute from our model for each EFM) and that a_y is constant (because we do not consider the effects of translational inhibitors). With these assumptions, the growth rate is given by

$$\mu = \frac{1}{\frac{1}{a_x} + \frac{1}{a_y}} \underbrace{[x + y]}_{c_{\mathrm{vp}}}, \tag{8}$$

where c_{vp} is the (constant) sum of variable protein fractions. We now set $a_x = v_{\mathrm{bm}}/c_{\mathrm{bm}}$, where v_{bm} is the biomass production rate (in carbon molar biomass / time) per metabolic enzyme (in carbon molar) and c_{bm} is the biomass concentration in cells (in carbon molar). The value of a_y can be obtained from proteomics data by a linear regression between growth rate and ribosome fraction. Eventually, we obtain

$$\mu = \frac{1}{\frac{c_{\mathrm{bm}}}{v_{\mathrm{bm}}} + \frac{1}{a_y}} \cdot c_{\mathrm{vp}} = \frac{c_{\mathrm{vp}} \cdot v_{\mathrm{bm}}}{c_{\mathrm{bm}} + \frac{1}{a_y}\, v_{\mathrm{bm}}}. \tag{9}$$

This is a hyperbolic function just like the one we derived before. By adjusting this formula to proteomics data, we would obtain the same parameters as above.

1.4 EFCM provides advantages over common kinetic or constraint-based modeling methods

By combining full information about kinetics and enzyme costs with a flux optimization approach, EFCM closes the gap between kinetic and stoichiometry-based models. It provides a clear theoretical link between kinetic models and existing network-based approaches which have incorporated some of the kinetic information. In contrast to these existing methods, EFCM allows for systematic studies of parameter sensitivities and uncertainties. Due to the screening of EFMs, its numerical effort is much higher. However, after one single calculation run, gene knockouts can be easily studied without any additional numerical effort. The main advantages over existing kinetic or constraint-based methods are as follows.

- **Advantage over a direct optimization of enzyme levels in kinetic models** Our optimization procedure is equivalent to a direct optimization of enzyme levels, which would often be numerically impossible: imagine that we treat the enzyme levels E_l in a kinetic model as free variables to be optimized for minimizing the ratio $v_{\mathrm{BM}}(\mathbf{E})/E_{\mathrm{met}}(\mathbf{E})$. This would be computationally costly: the objective function $v_{\mathrm{BM}}(\mathbf{E})/E_{\mathrm{met}}(\mathbf{E})$ may be difficult to evaluate (because this entails solving a kinetic model for its steady state), but is also likely to be non-convex and non-concave, with potentially many local minima. In EFCM, in contrast, all calculation steps are computationally tractable for medium-sized models. Another advantage of EFCM is that fluxes and metabolite constraints (e.g. stationarity, kinetics, thermodynamics, and physiological bounds) can be imposed easily. Moreover, it is instructive to consider the set of EFMs and to compute the growth rates even for the non-optimal ones. The rate/yield scatter plots show directly which EFMs become growth-optimal, under what conditions, and how the optimum flux mode can switch following parameter

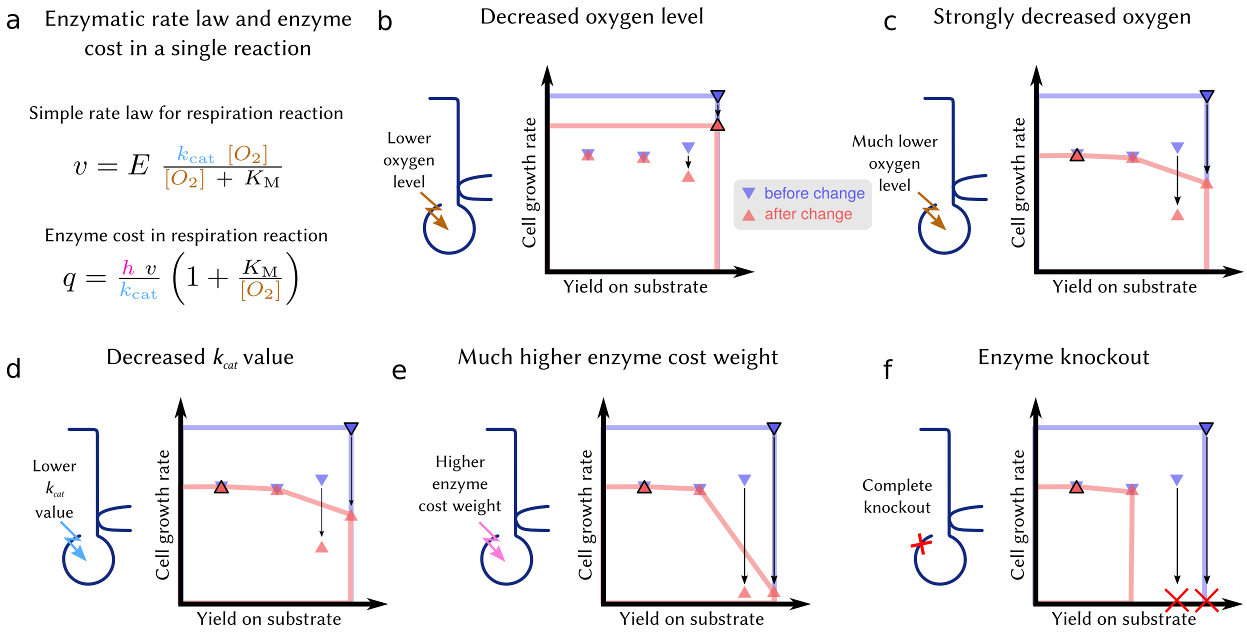

Figure S2: **EFCM can be used to simulate a wide range of enzyme perturbations and their effects on growth and metabolic strategies** (a) From an enzymatic rate law (in this schematic example, a simplified hypothetical Michaelis-Menten rate law with oxygen as a substrate), we obtain a formula for the enzyme cost as a function of flux, k_{cat} value, enzyme burden h, and oxygen level [O_2]. Changing the latter parameters will affect the enzyme cost (and therefore the growth rate) of each EFM differently. For example, EFMs that do not use the perturbed reaction will not be affected and their growth rates remain unchanged (in this case, these are the anaerobic EFMs). Panels (b-f) show, schematically, the changes in the rate/yield diagram resulting from different parameter perturbations. In each panel, the EFM with the highest growth rate is marked by a black frame. The blue and red polygons highlight the Pareto front before and after the change, respectively. If we lower the oxygen level (b), the growth-maximizing EFM shows a lower growth rate, but remains optimal. Only at much lower oxygen levels (c), another (oxygen-independent) EFM shows the highest growth rate. A similar change can be obtained by decreasing the k_{cat} value (d) or increasing the enzyme cost weight (e). (f) When an enzyme is knocked out, all EFMs that use this reaction become infeasible and disappear from the plot. The same effect could be reached by an extreme decrease in oxygen or k_{cat} value, or by an extreme increase in the enzyme cost weight.

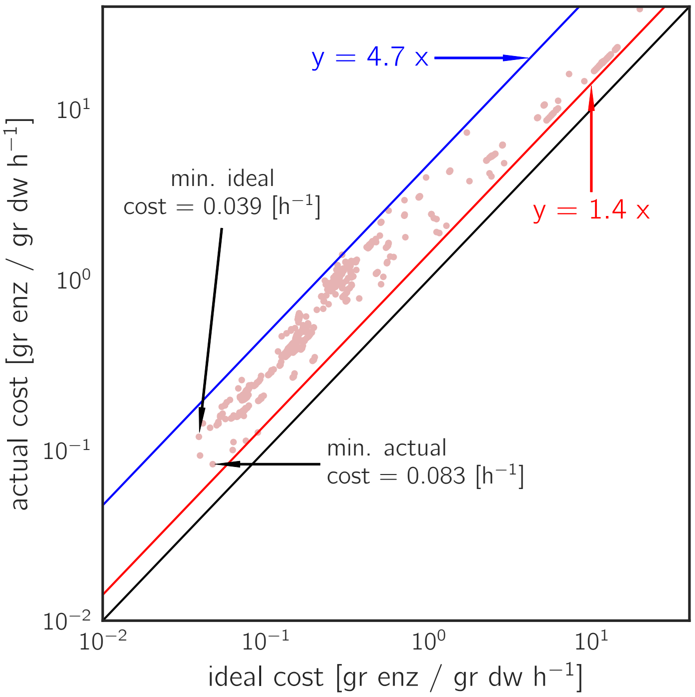

Figure S3: **Two estimates of the total enzyme cost.** The total enzyme cost, as computed by EFCM, can be approximated by assuming that all enzyme molecules work at their full capacity (i.e. full substrate saturation, no reverse fluxes, no product saturation). This simplification leads to the "ideal", capacity-based enzyme cost $q^{\rm cat} = \sum_i (|v_i| \cdot w_i)/k_{{\rm cat},i}$ [7]. Since $q^{\rm cat}$ yields a lower bound on the enzyme demand as calculated by EFCM ("actual cost", on the y-axis), all points in the plot must lie above the $y = x$ line (black). Actually, we find that all points lie even above the $y = 1.4x$ line (red), so the actual cost for our model in standard conditions (also for non-elementary flows) is at least 1.4 times higher than the ideal cost. Interestingly, the actual cost is also bounded from above by the $y = 4.7x$ line (blue). The two minimal-cost EFMs (i.e. the EFM with the lowest ideal cost and the EFM with the lowest actual cost) are indicated with arrows. Since the ideal cost can be calculated easily as a linear function of the flux, it can be used as an approximation of the actual cost in certain applications, e.g. to quickly discard EFMs that would lead to very low growth rates.

changes, including variable external conditions, changing enzyme parameters or cost weights, and enzyme knockouts (see Figure S2).

- **Advantage over constraint-based methods with linear flux cost functions** EFCM predicts metabolic fluxes and enzyme profiles *ab initio*, i.e. without requiring any conditions-specific measurements such as proteome or gene transcription data. Nevertheless, our calculations of growth rates rely on a large number of kinetic constants, and uncertainties in these parameters will introduce uncertainties into all our predictions. However, methods with fewer unknown parameters all have their own drawbacks. Stoichiometry-based methods (e.g. FBA without any additional flux constraints) do not require such parameters, but would not be able to address rate/yield trade-offs because their model assumptions force growth rates and yields to be proportional (see Figure S14). Our method can be compared to variants of Flux Balance Analysis that employ flux cost functions to mimic enzyme cost. For example, the total enzyme demand $E_{\rm tot} = \sum_l E_l$ of a pathway or network can be approximated by $E_{\rm tot} \approx E_{\rm lb} = \sum_l \frac{v_l}{k_{{\rm cat},l}}$, which puts a lower bound[1] on the true value of $E_{\rm tot}$. In EFCM, we could use this linear function $E_{\rm lb}(\mathbf{v})$ instead of the true enzyme cost derived from ECM[2]. However, the approximation would not only yield unrealistically low

[1]A similar idea underlies FBA with molecular crowding. In this method, the enzyme demand is not used as an objective to be minimized, but as a variable to be constrained during optimization. Fluxes are constrained by a bound $v_l \leq v_l^{\rm max} = E_l\, k_{{\rm cat},l}$ on every reaction flux. An upper limit on the sum of enzyme levels $E_{\rm tot,max} \geq E_{\rm tot} = \sum_l E_l$ represents the limited space available for enzymes.

[2]The resulting method would be equivalent to a flux balance analysis with fixed biomass production rate, no other flux constraints,

predictions of enzyme costs, but would do so to different extents across EFMs (see Figure S3). This would distort the prediction of growth-optimizing metabolic strategies.

1.5 A kinetic description of enzyme capacity utilization

Recent FBA methods, such as FBA with flux minimization [8], molecular crowding [9], or constrained allocations [10] address rate/yield trade-offs by bounding or minimizing the presumable enzyme demand. Such methods can predict low-yield flux modes, but they ignore the complex kinetic dependencies between reactions due to shared metabolites. As suggested by our EFCM results, enzymes may work below their maximal rates not because they are deliberately left unused, but because of the fact that enzymes require high substrate and low product levels inorder to be thermodynamically and kinetically efficient. This causes contradicting requirements in different reactions. Even in the best possible compromise, many enzymes will not be efficiently used. The assumption that some (or all) enzymes always operate at their maximal capacity would lead to an overestimation of growth rates. The difference between actual and predicted enzyme levels reflects, among other things, the fact that in each given moment, some enzyme molecules will not be processing a substrate molecule. In [11], these enzymes were called "unused enzyme fraction". As pointed out in the main text, we think that this term is misleading.

Nevertheless, to simplify EFCM, one could assume that enzymes work at a constant capacity utilization: in this case, fluxes and enzyme levels would not be related by rate laws, but by simple proportionality factors. For example, if we assumingd that all enzymes work at their maximal speed (as given by their k_{cat} values), the optimization of metabolite concentrations with respect to the total enyzme cost would become obsolete: using the enzyme molecular masses and k_{cat} values, we could directly translate any flux mode into a total required amount of enzyme by a simple linear formula, which does not depend on metabolite concentrations. This simplified version is used by satFBA [12], with the addition that the cost weights of the transport reactions can be varied to reflect differential saturation of the transporter enzymes, which allows for the investigation of changing external conditions (similar to our Figures S14(a-b) and S15).

In a previous article [7], we showed that the assumption of fully efficient enzymes yields inferior predictions for enzyme concentrations, and as expected, the growth rate prediction is harmed as well. The growth rate would be overestimated by a factor of about 2.4 (see Figure S3) and, more severely, the growth differences between EFMs would be distorted. This overestimation is purely an artifact and has no biological interpretation. Given the overestimation of the growth rate, it seems quite surprising that these methods can predict metabolic states quite effectively (e.g. [13]). In the case of Resource Balance Analysis (RBA) [14], the overestimation of growth rates is avoided by using experimentally measured *apparent* k_{cat} *values*, which are lower than the actual k_{cat} values and capture the fact that enzymes work below their full efficiency. In RBA simulations, where growth rate is a simulation parameter, different apparent k_{cat} values are chosen for different growth rates, reflecting the fact that enzyme efficiencies depend on metabolite levels [7], which vary between growth rates.

1.6 Extending EFCM to larger models and multiple enzyme cost functions

When extending EFCM to larger networks, one needs to address two main challenges: the numerical effort and data availability. Since the ECM problem for each EFM is convex, it may remain solvable even for large networks (as noted by e.g. [15]), but the EFMs of large networks would greatly increase in number. A subsampling of EFMs [16] could be problematic because, depending on model conditions, the high-growth EFMs may easily be missed (see section 3.1). A promising avenue is to subdivide large networks by defining

and a minimization of a weighted sum of fluxes, namely $E_{\mathrm{lb}}(\mathbf{v})$.

some key metabolites to be external [17] and controlling their concentrations. The resulting subnetworks can be analyzed independently [18], and their EFMs can be combined to yield favorable flux distributions. In any case, the second problem remains: when extending the network to more peripheral pathways, these pathways will be poorly covered with kinetic data, and the uncertainties about model parameters will increase. Such uncertainties may have a strong effect on the predictions, suggesting that simpler models that require fewer parameters would be preferable. Indeed, focusing only on core metabolism and lumping together some enzymatic complexes into single steps (such as the phosphotransferase system and the electron transport chain) was a deliberate decision that we took to reduce this parameter space. Possibly, our model could be further simplified without greatly affecting the results.

As another extension of EFCM, one could account for multiple cost functions. In our calculations, enzyme profiles were scored by total enzyme mass as a single cost. In a previous publication, EFMs had been scored by multiple cost terms describing different investments in enzyme production [19]. For each EFM, the required enzymes were scored by five different cost values: the investments in atomic carbon, nitrogen, and sulfur, the total protein chain length, and the total length of the DNA coding for these enzymes, and Pareto optimality was used to assess trade-offs between these costs. However, this previous work assessed only the qualitative proteome (i.e. the set of enzymes required for a given EFM), and not the quantitative enzyme levels. Building on [19], EFCM could be easily extended to incorporate multiple enzyme cost functions to be studied by Pareto optimality.

2 Model description

This section provides details about our *E. coli* model: its network structure, the choice of kinetic equations and enzyme parameters, the usage of physical units and the practical calculation of growth rates, the choice of external conditions, and some statistics about elementary flux modes.

2.1 Network structure

The network structure of our *E. coli* model (see Figure S4 and Table S2) is based on the model by Carlson (2004) [20]. For a kinetic model, we decided to split some linear chains of reactions, since each reaction in the chain might have different kinetics. Therefore, we changed several details of the model: we split the lumped reactions R7r, R10, R54r and R55r into separate reaction steps, we added the Entner-Doudoroff pathway (reactions R60 and R61r) and merged some reactions (the new reactions R27 and R27b) (see Table S3). We kept the ethanol (R90) and CO_2 (R97r) export in the model, but did not consider it in the kinetic calculation of enzyme cost because this is a passive, non-catalyzed process. Finally, we set the internal concentrations of CO_2 and ethanol to 1 mM, which we assumed to be sufficient to allow for an export flux through diffusion. The stoichiometric coefficients for the biomass reaction (R70) were taken from the original model (Table II in [20] for a doubling time of 30 minutes). We added the measured maintenance flux to the ATP stoichiometry in the biomass equation, which changed the stoichiometry constant for ATP to -1641. An SBML version of the model is provided in the Supplementary Files. The EFMs were calculated with EFMtool [21]. We scaled all EFMs to a standard biomass production of 1. The model contains a total of 2772 EFMs, out of which 1566 produce biomass. 760 of the biomass-producing EFMs are not feasible because they both need oxygen and use an oxygen sensitive reaction, 97 are facultatively aerobic, 470 are strictly aerobic, and 239 are strictly anaerobic (see Figure 2(b)). To avoid biases in our growth predictions, we considered all EFMs that have a non-zero biomass yield, even those that contain physiologically unreasonable fluxes. For example, the futile cycling between PEP and pyruvate (by the combined activity of pyruvate kinase (**pyk**)

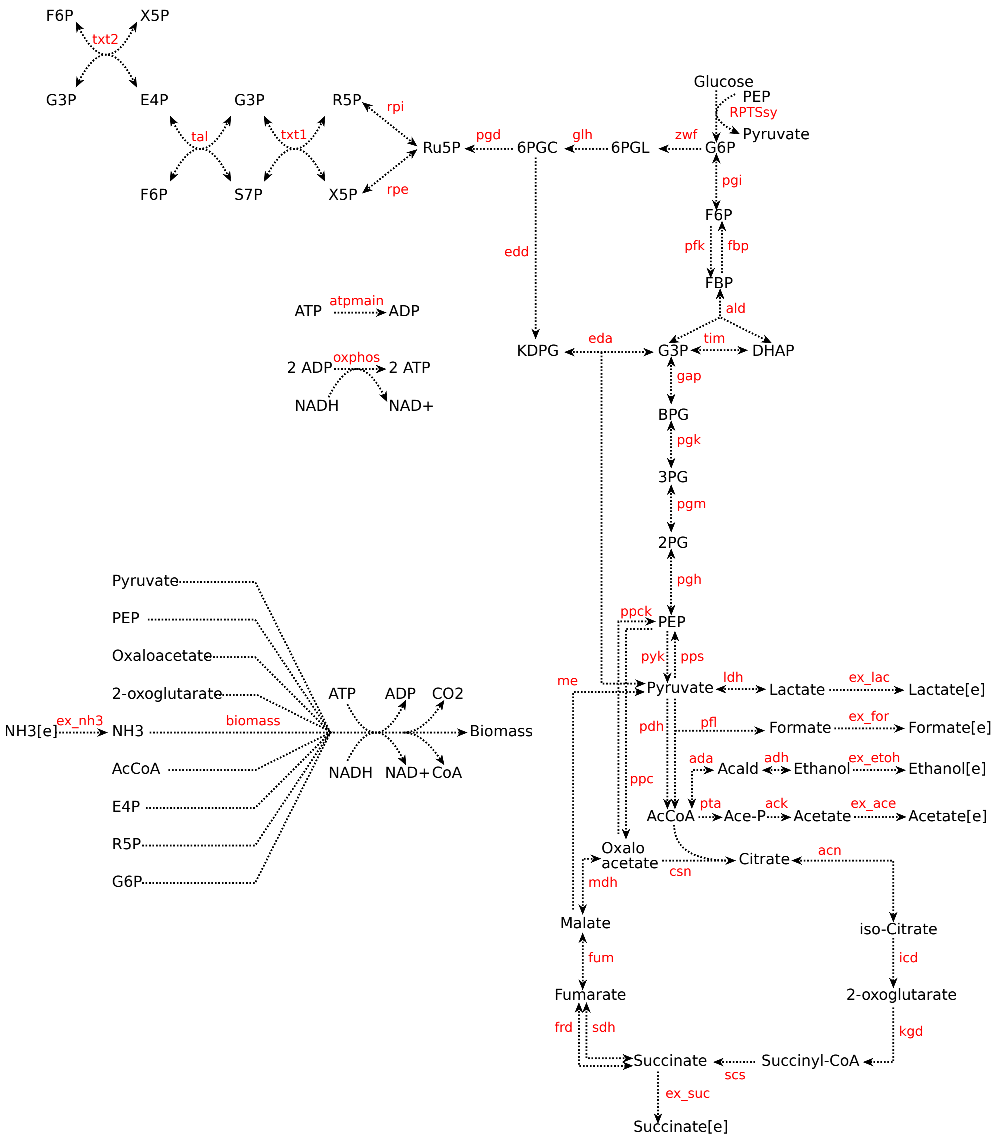

Figure S4: **The reaction network of core carbon metabolism in *E. coli*.**

and pyruvate water dikinase (**pps**)) wastes ATP and can be expected to be suppressed by strict enzyme regulation [22]. Such EFMs were consistently predicted to cause low growth rates and had no effect on the outcomes of our study. Some statistics about the biomass-producing EFMs can be found in Figure S7.

2.2 Kinetic equations

We used the same type of rate equations for all reactions [3]. Reversible reactions are marked by an "r" after the reaction number (see Table S2). Reversible (v_r) and irreversible (v_i) reactions are modeled as follows:

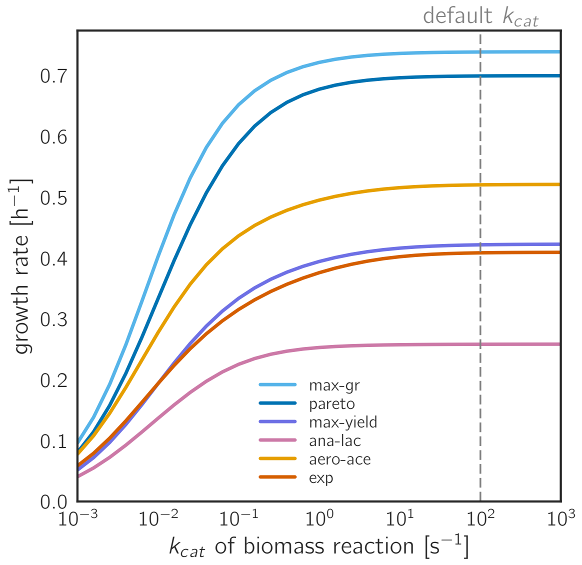

Figure S5: **Growth rates of the focal EFMs, depending on the k_{cat} value of the biomass reaction (R70).** In all cases, growth rates increase with the maximal velocity of the biomass reaction. Since kinetics and enyzme demands of the biomass reaction are hard to determine, we chose a high k_{cat} value (dotted line) as a standard parameter. With this value, the enzyme cost of the biomass reaction is negligible and the maximal growth rates are determined by metabolism (and different between EFMs) rather than by biomass production itself. The negligible enzyme cost agrees with our definition of "metabolic enzymes within the proteome" which excludes all proteins involved in macromolecule production.

$$v_r = e_r \cdot k_{\text{cat},r} \frac{\prod_j \left(\frac{s_j}{K_{M,s_j,r}}\right)^{n_j} \left(1 - \frac{\prod_k p_k^{n_k} / \prod_j s_j^{n_j}}{K_{\text{eq,r}}}\right)}{\prod_j \left(1 + \frac{s_j}{K_{M,s_j,r}}\right)^{n_j} + \prod_k \left(1 + \frac{p_k}{K_{M,p_k,r}}\right)^{n_k} - 1} \tag{10}$$

$$v_i = e_i \cdot k_{\text{cat},i} \frac{\prod_j \left(\frac{s_j}{K_{M,s_j,i}}\right)^{n_j}}{\prod_j \left(1 + \frac{s_j}{K_{M,s_j,i}}\right)^{n_j} + \prod_k \left(1 + \frac{p_k}{K_{M,p_k,i}}\right)^{n_k} - 1}, \tag{11}$$

with enzyme concentration e_i, substrate concentrations s_j, product concentrations p_k, and (absolute value of the) stoichiometric coefficient n. The K_M values are the Michaelis-Menten constants, the k_{cat} values the turnover numbers, and the K_{eq} values the equilibrium constants. In our model, macromolecule production is quantified by a single biomass production rate v_{BM}. The biomass reaction is a lumped reaction of all processes involved in biomass production, and its rate law represents the action of many (not explicitly modeled) cellular processes operating in steady state [23]:

$$v_{\text{biom}} = \frac{e_{\text{biom}} \cdot k_{\text{cat,}}^{\text{biom}}}{1 + \sum_j \frac{K_{M,\text{s}_\text{j},}^{\text{biom}}}{s_j}} \tag{12}$$

Equation (12) corresponds to the equation given in [23] with a single unit in the template ($n = 1$) and e_{biom} as the template concentration. The s_j are the substrate levels of the biomass reaction and $K_{M,s_j,biom}$ their Michaelis-Menten constants. Since this reaction is a lumped reaction that summarizes a wide range of biosynthetic reactions, its catalytic constant does not have a direct biochemical meaning. We thus opted not to give too much cost weight to the biomass production and chose a k_{cat} value that does not affect the rate very much (see Figure S5 and compare with Figure S20). With the catalytic constant chosen, the kinetics of the biomass reaction does not limit growth, i.e. growth control is only exerted by the other, metabolic reactions.

2.3 Choice of consistent model parameters by parameter balancing

Parameter balancing [1] was used to translate measured kinetic constants from the literature into a complete and consistent set of model parameters ($k_{\rm cat}$, $K_{\rm eq}$, and $K_{\rm M}$ values). Unknown $k_{\rm cat}$ values, for example, were substituted by values around 100 s^{-1}, adjusted to satisfy Haldane relationships, i.e. the thermodynamics-based laws that link them to $K_{\rm M}$ values and equilibrium constants. Plausible parameter ranges were used to define prior distributions (e.g. a mean value and a standard deviation for logarithmic $k_{\rm cat}$ values). The median value of 100 s^{-1} was chosen because $k_{\rm cat}$ values in core metabolism tend to be higher than in metabolism in general (typical value around 10 s^{-1}) [24]. The input and output files of parameter balancing can be found in the Supplementary Files. The kinetic input data were obtained from the literature (see Supplementary Files). Some $k_{\rm cat}$ values were calculated from specific activities (SA) with the formula $k_{\rm cat} = {\rm SA} \cdot {\rm MW(in\ kDalton)}/(60 \cdot {\rm nr}_{\rm catalyticsites})$. The weights were obtained by calculating the molecular masses for enzyme complexes and dividing by the number of catalytic sites. Whenever the number of catalytic sites was not known, we used the number of subunits as a proxy for the number of catalytic sites. Since no kinetic information was available for the biomass equation, we set the K_M values to the measured intracellular concentrations when available, and otherwise to a low value as not to influence the results (see Supplementary Files). Overall, we found literature values for the $k_{\rm cat}$ of 26 out of 51 reactions, and $K_{\rm M}$ values for 87 out of the 171 reactant-enzyme pairs (i.e. about 50% coverage in both cases). The parameter values after balancing, which are used in the paper, can be found in Tables S5 ($k_{\rm cat}$ values), S6 (K_M values) and S7 (K_{eq} values).

2.4 Calculation of specific growth rate and yield

To calculate the cell growth rate μ (using formulae from Equations (4.6)–(7)), we first translate the biomass flux to actual mass units (e.g. grams) by summing up the molecular masses of the biomass reactants, multiplied by their stoichiometric coefficient. The ATP/ADP and NADH/NAD$^+$ cofactor pairs are ignored in this calculation because they produce a negligible amount of biomass. That means that one mole of biomass weighs about 20.7 kg. Details of the mass calculation of the biomass are given in Table S4. Then, we convert the biomass flux v_{R70} in the model to the biomass production rate $v_{\rm BM}$ in the growth equations:

$$\begin{aligned} v_{\rm BM} &= v_{R70}\ [{\rm mM\ s^{-1}}] \cdot 2.07 \times 10^4\ [{\rm mg\ mmol^{-1}}] \cdot 3.6 \times 10^3\ [{\rm s\ h^{-1}}] \\ &= v_{R70} \cdot 7.45 \times 10^7\ [{\rm mg\ s\ mmol^{-1}\ h^{-1}}]. \end{aligned} \quad (13)$$

Our model calculates the abundance of each enzyme (e_i, in mM) required for realizing this biomass flux. To obtain the total enzyme mass required, we multiply each e_i by the enzyme's molecular mass per active site (w_i, given in [mg mmol^{-1}]). Therefore, the total enzyme cost will be $E_{\rm met} = \sum_i e_i w_i$ [mg l^{-1}]. Finally, the *enzyme doubling time* is given by

$$\begin{aligned} \tau_{\rm met} &= \frac{\ln(2) E_{\rm met}}{v_{\rm BM}} \quad (14) \\ &= v_{R70}^{-1} \cdot 9.3 \times 10^{-9} [{\rm mmol\ h\ mg^{-1}\ s^{-1}}] \cdot E_{\rm met}. \end{aligned}$$

According to Equation (7), the doubling time of the cell reads

$$\begin{aligned} T &= 7.4\ \tau_{\rm met} + 0.51[{\rm h}] \quad (15) \\ &= \frac{1}{v_{R70}} \cdot 6.9 \times 10^{-8}\ [{\rm mg\ s\ mmol^{-1}\ h^{-1}}] \cdot E_{\rm met} + 0.51[{\rm h}]. \end{aligned}$$

From Equations (4.6), (4) and (13), the growth rate μ can be calculated from total enzyme cost E_{met} and from biomass flux v_{R70} with the following formula:

$$\mu \;=\; \frac{v_{R70} \cdot 10^7 \text{ [mg s mmol}^{-1}\text{ h}^{-1}]}{E_{\text{met}} + v_{R70} \cdot 7.5 \times 10^6 \text{ [mg s mmol}^{-1}]}. \tag{16}$$

The biomass yield is expressed in milligram biomass per millimole of carbon atoms uptake. Therefore, we need to convert biomass production to grams and substrate uptake rates to mole carbons. Since glucose molecules contain six carbon atoms, and a mole of biomass weighs 20666 grams (see Table S4), the biomass yield is given by

$$\begin{aligned} Y_{\text{g/C}} &= \frac{20666 \text{ [mg mmol}^{-1}]\, v_{R70}}{6 \cdot v_{\text{pts}}} \\ &= \frac{v_{R70}}{v_{\text{pts}}} \cdot 3.4 \times 10^3 \text{ [mg mmol}^{-1}], \end{aligned} \tag{17}$$

where v_{pts} is the flux in the PTS glucose uptake system (reaction **R1** in the model).

2.5 Absolute values for enzyme concentrations

Our model predicts enzyme concentrations abundances in mM, but initially these values are not properly scaled. To make all EFMs comparable, we fix the biomass production rate v_{R70} at a standard value of $1 \text{ [mM s}^{-1}]$. Thus, before comparing the predicted enzyme levels with data, we need to compute our hidden scaling factor, the absolute value of v_{BM}. An average bacterial cell consists of about 30% dry matter [25], which translates to a dry density of $\rho \approx 3 \times 10^5 \text{ [mg l}^{-1}]$. A typical value for the growth rate would be $\mu \approx 1 \text{ [h}^{-1}]$, which would be enough to for a rough estimate of the biomass rate, given by $v_{\text{BM}} = \rho \cdot \mu$. Using equation (13) we now obtain

$$\begin{aligned} v_{R70} &= \frac{v_{\text{BM}} \text{ [mg l}^{-1}\text{ h}^{-1}]}{7.45 \cdot 10^7 \text{ [mg s mmol}^{-1}\text{ h}^{-1}]} \\ &= \frac{\rho \text{ [mg l}^{-1}] \cdot \mu \text{ [h}^{-1}]}{7.45 \cdot 10^7 \text{ [mg s mmol}^{-1}\text{ h}^{-1}]} \\ &\approx 4 \times 10^{-3} \text{ [mM s}^{-1}]\,. \end{aligned} \tag{18}$$

This result means that our standard value for v_{R70} is ∼250 times too high, and so are the nominal estimates for enzyme concentrations. To obtain realistic estimated values, one must therefore divide each e_i by 250.

It is important to note that a wrong scaling of v_{R70} and e_i does not affect the predictions about growth rate and yield. One can see that in formulae (2.4) and (2.4) this scaling factor affects both the numerators and the denominators and therefore cancels out. This is not a coincidence, but a feature of the way we calculate growth rate, namely by dividing the rate of biomass production by the required enzyme amount for that specific rate. As an outcome of this independence, one can also use the predicted growth rate directly in equation (18) instead of the unit value ($1 \text{ [h}^{-1}]$), even though the value of v_{R70} and the enzyme concentrations were used to calculate the growth rate in the first place.

2.6 Choice of standard external conditions

As a standard condition for our simulated cells, we chose a high external glucose concentration of 100 mM. For oxygen we chose the concentration from the same paper as we used for the kinetics of the oxygen-using

reactions [26], namely 0.21 mM. The concentration of ammonia (NH_3), another external compound taken up, was set to 10 times more than the highest K_M to ensure saturation (1.0 mM). The levels of excreted metabolites were assumed to be low and were set to 0.01 mM, except for ethanol and CO_2 which were set to 1 mM (which are actually internal metabolites, since we treat the export reactions as non-enzymatic).

2.7 Details on the elementary flux modes

Some biological and statistical properties of the EFMs are shown in Figures S6, S7, S8, and S9.

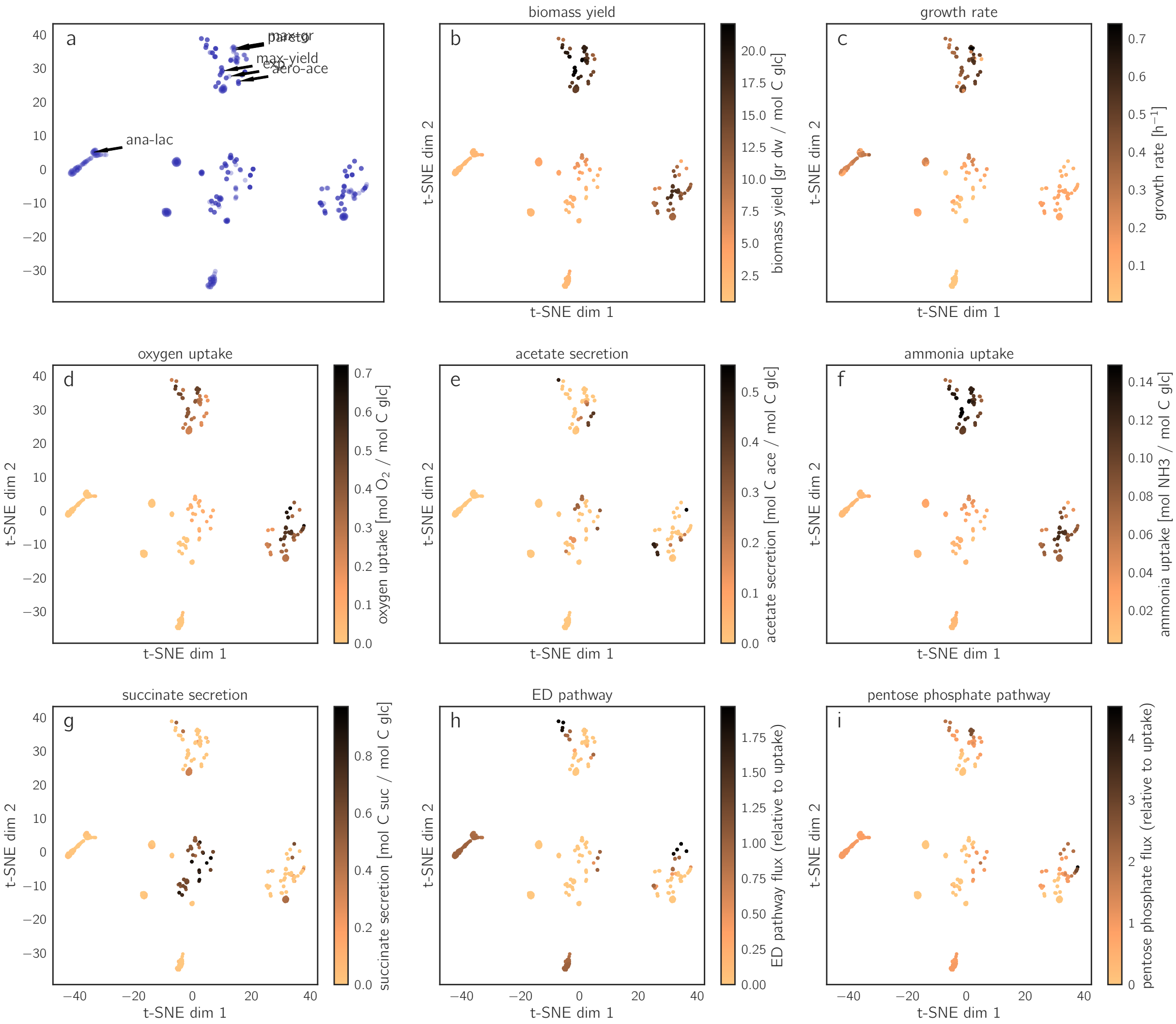

Figure S6: **Elementary flux modes, visualized using the t-SNE algorithm.** (a) Elementary flux modes are vectors in a high-dimensional flux space. The t-SNE algorithm [27] represents EFMs by points on a two-dimensional plane. It tries to preserve their original distances (i.e. the Euclidean distances in flux space) while spreading the points evenly over the plane. The biomass yield (b) and specific growth rate (c) are represented by a color scale. As expected, similar EFMs have very similar yields (since the yield is one of the dimensions in the flux space), but growth rates can significantly change between neighboring EFMs. In each of the panels (d-i) we show a single feature in color coding, all on the same EFM map. The t-SNE algorithm produces clusters of EFMs related to major uptake and secretion fluxes. Interestingly, all high-growth EFMs are contained in a single cluster (see (c)), even though t-SNE uses no information about enzyme kinetics or our standard growth conditions.

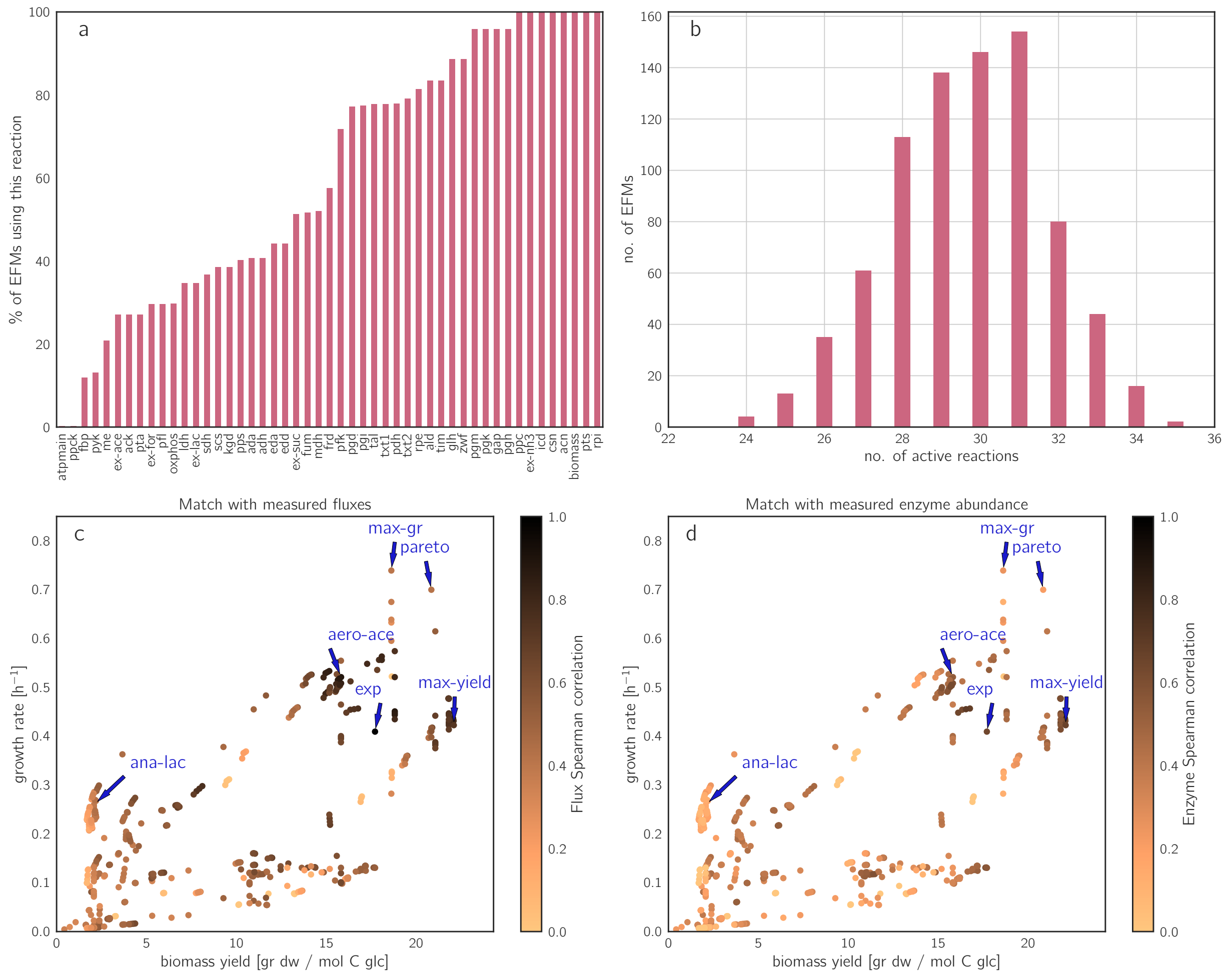

Figure S7: **Statistical properties of biomass-producing EFMs and correspondance between EFMs and the measured fluxes** (a) Usage of individual reactions by biomass-producing EFMs. For each reaction, a bar shows in what percentage of all EFMs this reaction is active. A core of 8 essential reactions is active in all biomass-producing EFMs (**ppc**, **ex-nh3**, **icd**, **csn**, **acn**, **biomass**, **pts**, and **rpi**). (b) Size distribution of biomass-producing EFMs (number of active reactions). (c) Similarity between EFMs and the measured flux distribution. The color of each EFM in this rate/yield plot corresponds to the Spearman correlation between the fluxes in that EFM and the experimentally measured fluxes from [28]. (d) Similarly, we plot the Spearman correlation between the estimated enzyme levels for each EFM and the measured enzyme levels from [29]. Note that even the point corresponding to *exp* does not have a correlation of 1, since, even though the fluxes are taken from experiments, the estimated enzyme abundances are still given by the ECM algorithm. Nevertheless, *exp* is among the EFMs with the highest correlations.

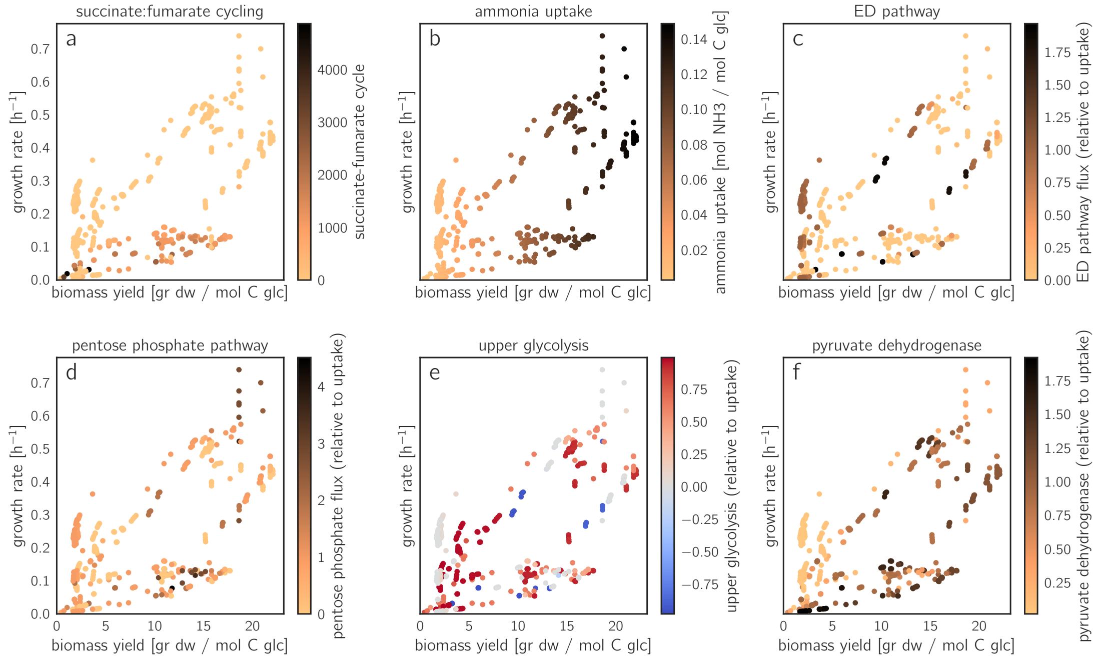

Figure S8: **Reaction fluxes in different EFMs.** Fluxes are plotted in a rate/yield diagram under standard conditions. More fluxes are given in main text Figure 3. (a) Succinate:fumerate cycling is not very beneficial and occurs only in suboptimal EFMs. (b) Ammonia uptake is strongly correlated with yield because ammonia directly enters the biomass reaction. (c) The ED pathway is consistently used by a group of low yield/high growth rate EFMs, but also in other parts of the spectrum. (d) The pentose phosphate pathway is also used throughout, but more in the higher-growth-rate EFMs. (e) The flux in upper glycolysis is reversible and therefore depicted using a red-blue colormap. It is zero for the seven EFMs with the highest growth rate and otherwise usually positive. Only a few EFMs with medium-low growth rates use the reverse direction. (f) The flux through pyruvate dehydrogenase, a large (and therefore costly) enzyme complex, is relatively low for the fastest EFMs.

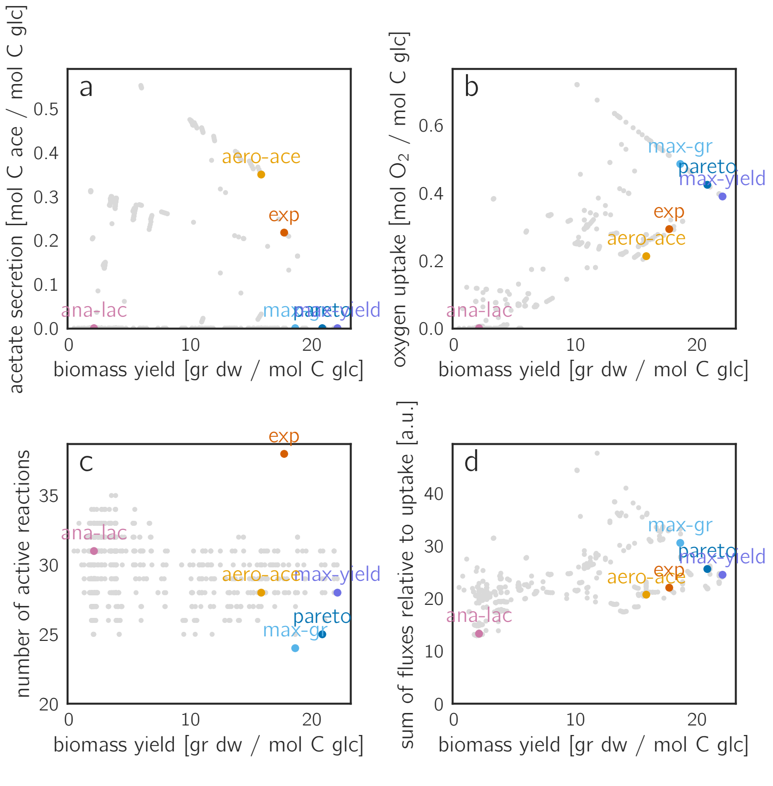

Figure S9: **Correlations of biomass yield with acetate flux, oxygen flux, number of reactions, and flux sum** All five quantities follow from the shapes of EFMs, independently of enzyme kinetics. (a) Acetate secretion, scaled by glucose uptake. Among the acetate-secreting EFMs, higher acetate secretion tends to imply lower biomass yields. (b) An oxygen uptake around 0.4 [mol O_2 per mol C glucose] maximizes the yield: a lower O_2 uptake implies higher by-product secretion (to make up for the ATP requirements), while at higher uptake rates, more carbon is oxidized and released as CO_2. (c) The number of active reactions, a simple measure of enzyme demand, shows little correlation with biomass yield. (d) The same holds for the sum of fluxes, scaled by the glucose uptake rate.

3 Model results

This section provides additional results on the predicted growth rates for different EFMs, under different external conditions (variation of glucose and oxygen levels), different choice of kinetic constants, a restricted usage of pathways, and enzyme knockouts. It also provides results on the necessary enzyme investments (for different EFMs, and under varying external conditions) and on the optimal metabolic strategies in chemostats at different growth rates (as determined from predicted Monod curve parameters).

3.1 Growth rates achieved by elementary flux modes

As shown in Figure S10, the predicted cell growth rates do not only vary widely across EFMs, but also show statistical distribution that assume very different shapes depending on biochemical external conditions. At standard (high oxygen) conditions, growth rates are relatively evenly distributed, while under low-oxygen conditions, a large number of EFMs (the oxygen-dependent ones) show very low growth rates, and only a very small percentage comes close to the maximal growth rate. This has practical consequences for modeling: since the number of EFMs can be large, it might be tempting to sample EFMs instead of enumerating them exhaustively, in the hope to find at least some EFMs with high growth rates. However, this approach would have failed in the low-oxygen case, because almost all EFMs yield very unfavorable growth rates. In this specific example, we might try to sample EFMs in such a way that oxygen-dependent EFMs are automatically discarded. However, in general cases, such heuristics may be hard to find. We conclude that a sampling of EFMs may yield some well-performing EFMs (as here, in high-oxygen conditions), but it may also fail (as here, in low-oxygen conditions).

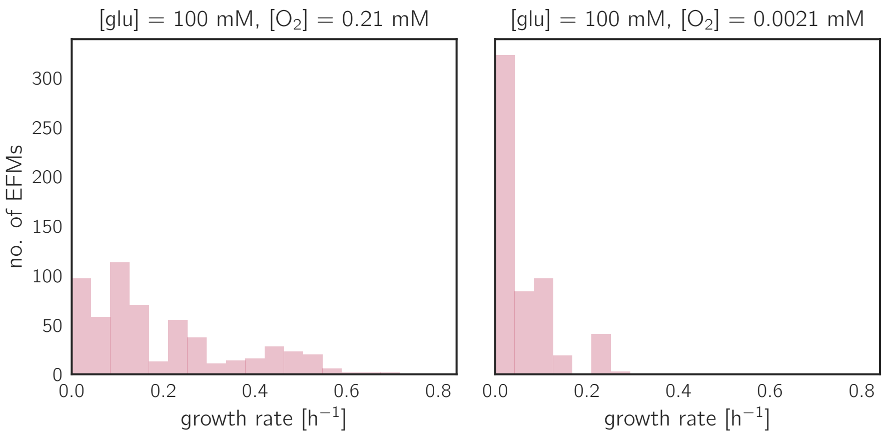

Figure S10: **Distribution of growth rates over EFMs.** (a) Under standard conditions, the growth rates are relatively evenly distributed, and a randomly sampling of EFMs is likely to yield some EFMs with high growth rates. (b) Under low-oxygen conditions, in contrast, the distribution is strongly skewed to the left. The chances of finding an EFM with a high growth rate (i.e. > 0.2 [h^{-1}]) by chance are very small.

3.2 Effect of individual enzyme parameters on cell growth

If we decrease the catalytic constant of an enzyme, all EFMs using this enzyme will show lower growth rates because the lower catalytic efficiency must be compensated by higher enzyme levels to maintain the flux in the EFM (see proof in SI section 4.5); as a consequence, the biomass production per enzyme investment decreases, and so does the growth rate. Figure S20 shows the effects of a simulated change in the catalytic

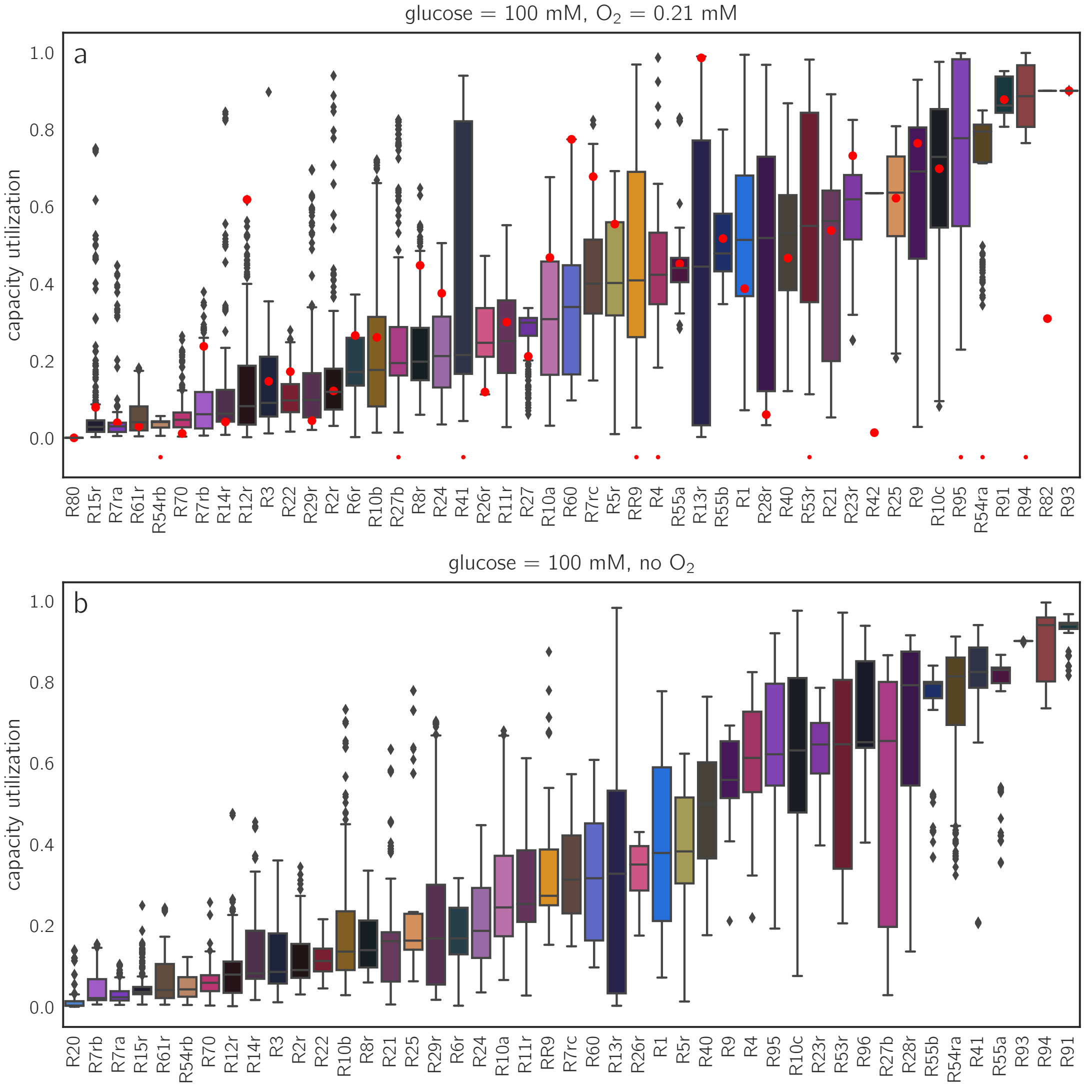

Figure S11: **Capacity utilization of enzymes in aerobic and anaerobic conditions.** (a) Capacity utilization in standard (high-oxygen) conditions. The box plot shows, for each enzyme, the range of capacity utilization values across EFMs. The capacity utilization is defined as the actual flux, divided by the "ideal" flux achievable under conditions of full substrate saturation and maximal thermodynamic driving force. The inter-quartile ranges (box sizes) are relatively small, showing that most enzymes have their typical capacity utilization values. These typical values, however, span almost the entire range between 0 and 1. In the plot, enzymes are sorted by their median values. Box colors correspond to other plots in this paper. Red dots denote the calculated capacity utilization for measured wild-type fluxes. (b) Capacity utilization for anaerobic conditions. The values for individual enzymes differ substantially from the values in (a).

constant of *tpi*, the triose-phosphate isomerase. Compared to standard conditions (light grey dots, same as in main text Figure 2(b)), the Pareto front now contains a few EFMs with similar yields, but very different growth rates. The growth rate of *max-yield*, which uses *tpi*, is greatly reduced, while the *max-gr* EFM is not affected since it has no flux through *tpi* at all. The sensitivities shown in Figure S20 (b) correspond to the curve slopes in Figure S20 (c) at the standard k_{cat} value. The grey shading marks the range of a two-fold increase or decrease around the standard k_{cat}. One can see that the linear approximation is not suitable for much larger changes in k_{cat}, such as the change shown in panel (a) (i.e. a 1000-fold decrease to "low k_{cat}"). The curves in Figure S20(c) are based on a global enzyme re-optimization after parameter changes. Therefore, if we focus on small parameter changes (which allow us to ignore effects higher than first order), the parameter sensitivities can be easily computed. The calculation of sensitivities for k_{cat} values,

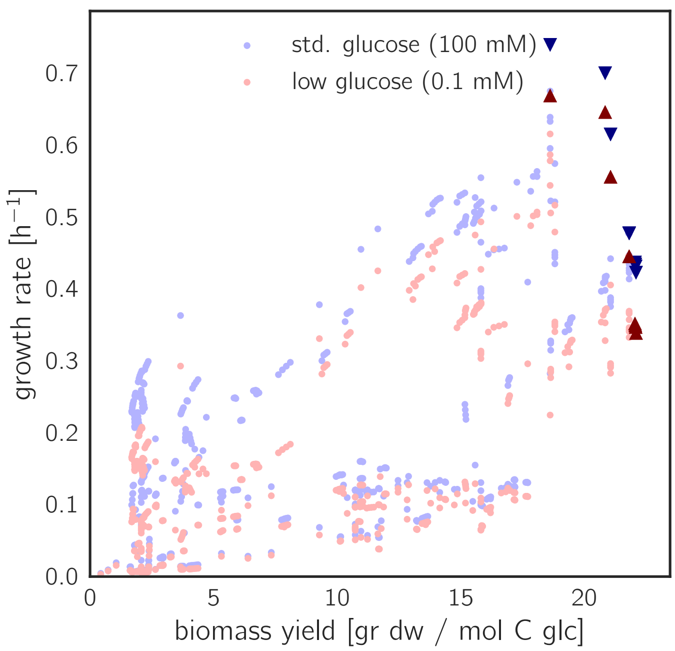

Figure S12: **The effect of a lower glucose level on growth.** A drop in glucose level (from 100 mM to 0.1 mM) decreases the growth rates of all EFMs, but to different extents. EFMs with low yields are more strongly affected (*ana-lac*, for example), since the relative glucose uptake rate, and therefore the enzyme burden of the PTS system, is larger. However, the Pareto front changes only slightly, and still consists of a few EFMs (Pareto optimal EFMs are marked by dark triangles).

equilbrium constants, and $K_{\rm M}$ values from previously computed optimal enzyme profile for an EFM under standard conditions is described in SI sections 4.2 and 4.3.

3.3 Monod curves and optimal EFMs under high-glucose or low-glucose conditions

In a glucose-limited chemostat, different dilution rates will lead to different cell densities and external glucose concentrations. Can we expect that cells use the same metabolic strategies in a wide range of growth rates, or with some strategies perform better at low growth rates and others perform better at high growth rates? Since our model uses glucose concentration as a parameter, EFCM can be used to answer such questions; whereas methods like classical FBA, in which all fluxes scale linearly with the glucose uptake flux, would not be applicable (see Figure S14). To study trade-offs between growth at low and high glucose levels, we first calculated a Monod curve for each individual EFM, assuming a wide range of external glucose concentrations. The Monod curve is typically characterized by the formula $\mu = \mu^{\max}\frac{[S]^h}{[S]^h+K_{\rm S}^h}$, where $\mu^{\max}$ is the maximal growth rate, $[S]$ is the concentration of the limiting nutrient (i.e. glucose) and $K_{\rm S}$ is the substrate saturation constant (or "Monod coefficient"). The Monod coefficient denotes the concentration of glucose at which the half-maximal growth rate is reached ($\mu = \frac{1}{2}\mu^{\max}$), and its reciprocal value can be seen as the cell's overall affinity for glucose. In a chemostat at high dilution rates, cells must grow fast because they can only survive if their maximal growth rate exceeds the dilution rate. At low dilution rates, in contrast, the higher cell density leads to very low glucose levels; in this case, there is a selection for cells that can grow fast at low glucose levels, typically cells with a low Monod constant.

Having computed the growth rates of all EFMs in a wide range of glucose concentrations, either in aerobic or anaerobic conditions, we determined the curve parameters for each EFM (Monod coefficient and maximal growth rate) by fitting the growth curve with a Hill function. Plotting $\mu^{\max}$ versus the affinity $1/K_{\rm S}$ and under aerobic conditions, we do find a trade-off (Figure S19(a)), and under anaerobic conditions, the trade-off develops becomes more pronounced (Figure S19(e)). In fact, the growth at low glucose concentration depends both on the Monod constant and on the maximal growth rate; as shown in Figure S19(i), our

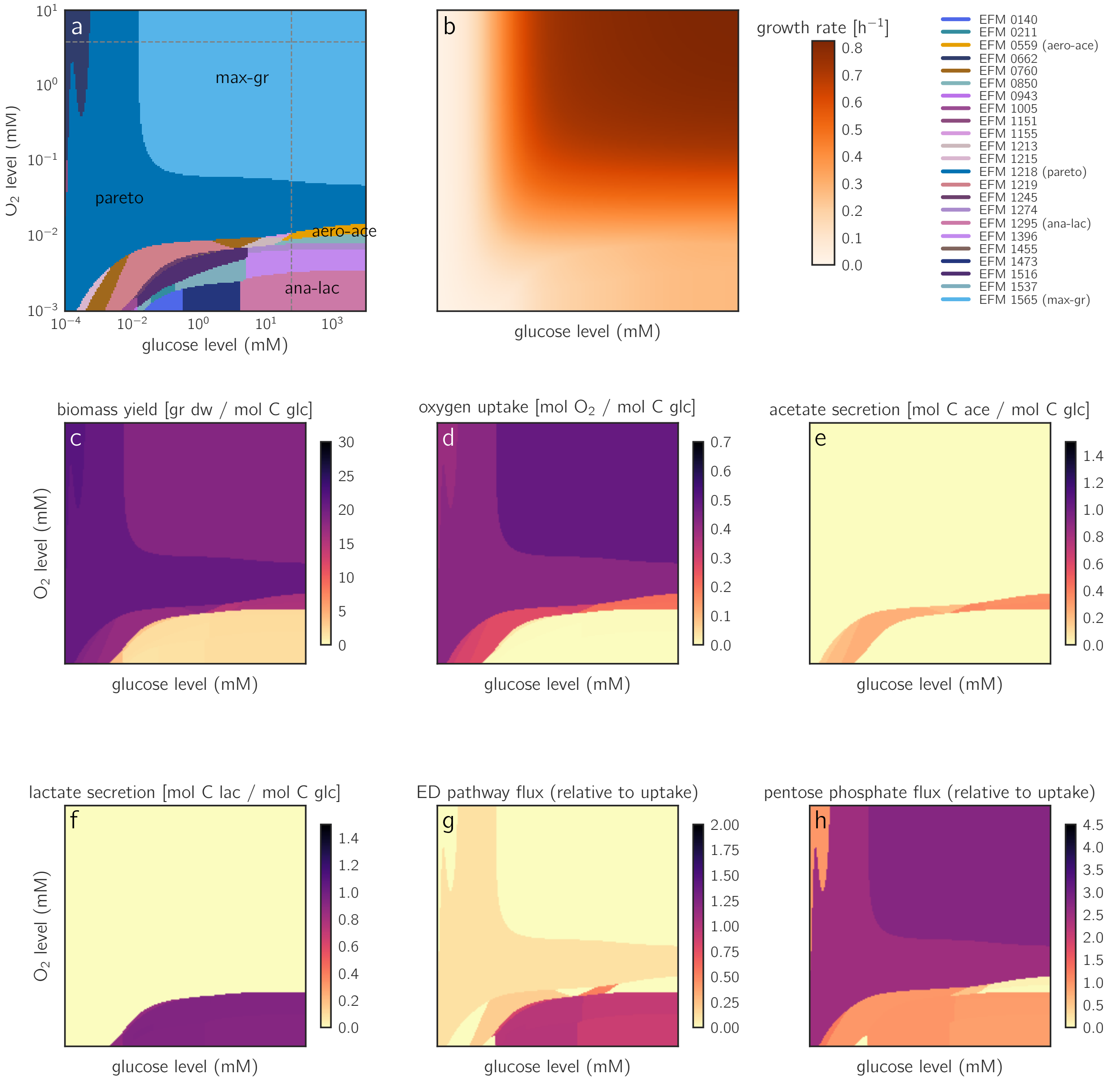

Figure S13: **Cell growth rate as a function of external glucose and oxygen levels.** For each combination of glucose and oxygen levels, maximal growth is realized by one optimal EFM (marked by colors). (a) 23 of the EFMs turn out to be optimal (i.e. have the highest growth rate) at least in some region of the glucose/oxygen plane. (b) Despite the complicated pattern of "winning" EFMs, the optimal growth rate changes smoothly with the glucose and oxygen levels. It is difficult to see any transitions except for one boundary that curves up around the lower right corner. In a chemostat experiment, an imposed growth rate could be realized by various combinations of external glucose and oxygen levels. Which of these combinations arises in the medium depends on the ratio of glucose and oxygen supplies to the chemostat. By plotting different EFM properties (c-h) of the optimal EFMs in this plane, we can see that only anaerobic EFMs are optimal in the low oxygen/high glucose region (d). Interestingly, acetate fermentation is only favorable in a narrow band around this anaerobic region and becomes unfavorable at higher O_2 levels (e).

simulations predict almost no trade-off as long as oxygen levels are high. In anaerobic conditions (Figure S19(m)), the trade-off becomes more pronounced, suggesting that the winning strategies depend on the dilution rate.

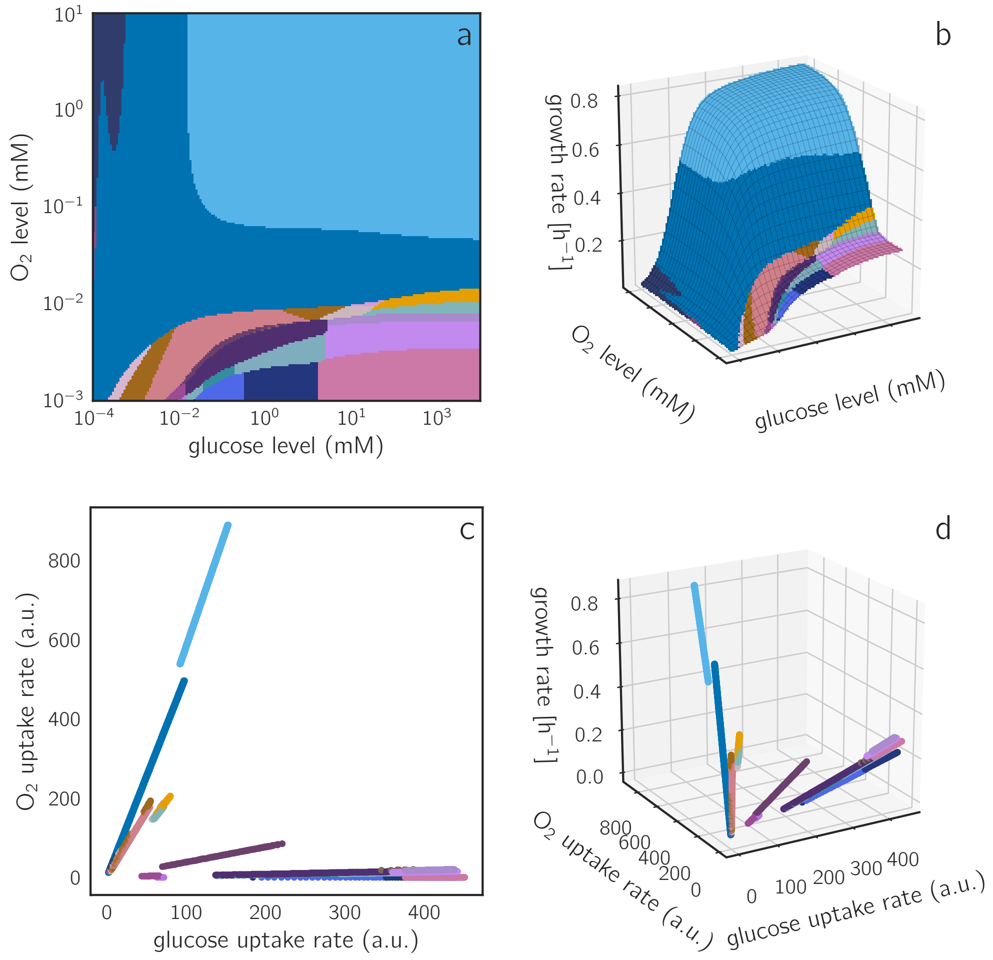

Figure S14: **Optimal EFMs depending on glucose and oxygen concentrations or uptake rates.** (a) The optimal EFMs across the glucose/oxygen plane (same figure as S13(a)). (b) The optimal EFM colors overlaid on a 3D surface plot, where height represents the optimal growth rate reached. (c) By changing the axes in panel (a) to the glucose and oxygen uptake rates, we obtain a very different picture. Due to our model assumptions, only EFMs can maximize the growth rate at a given condition, and since each EFM defines a constant ratio of glucose to oxygen uptake, its points are all positioned along a straight line (scaled by the growth rate). Therefore, this scatter plot is very sparse. (d) the same data as in (c), shown as a 3D plot where the z-axis is growth rate. Again, the points of each EFM are aligned since all rates scale linearly with the growth rate.

3.4 Growth of strains deficient in EMP glycolysis, ED glycolysis, or respiration

Our analysis of growth-yield trade-offs extends an earlier study of cost/yield trade-offs in ATP production. Flamholz *et al.* had studied cost-yield trade-offs between two variants of glycolysis, the Embden-Meyerhof-Parnas (EMP) and the Entner-Doudoroff (ED) pathway [30]. Their model comprised only glycolysis, and ATP yield and enzyme cost were compared between the two pathway variants at a fixed glucose uptake rate. The EMP pathway provides a two-fold yield, but requires even higher amounts of protein per ATP production flux. This leads to a cost-yield trade-off. The authors concluded that, under a high demand for ATP produced in glycolysis, cells should use the EMP pathway, while cells that have other "cheap" sources of ATP (e.g. photosynthesis) should rather use the ED pathway.

With our model, we can now study the choice between pathways more generally. Compared to the glycolysis model in [30], our model has the following additional features: (i) the model includes the TCA cycle and respiration, so choices between fermentation and respiration and choices between EMP and ED pathways

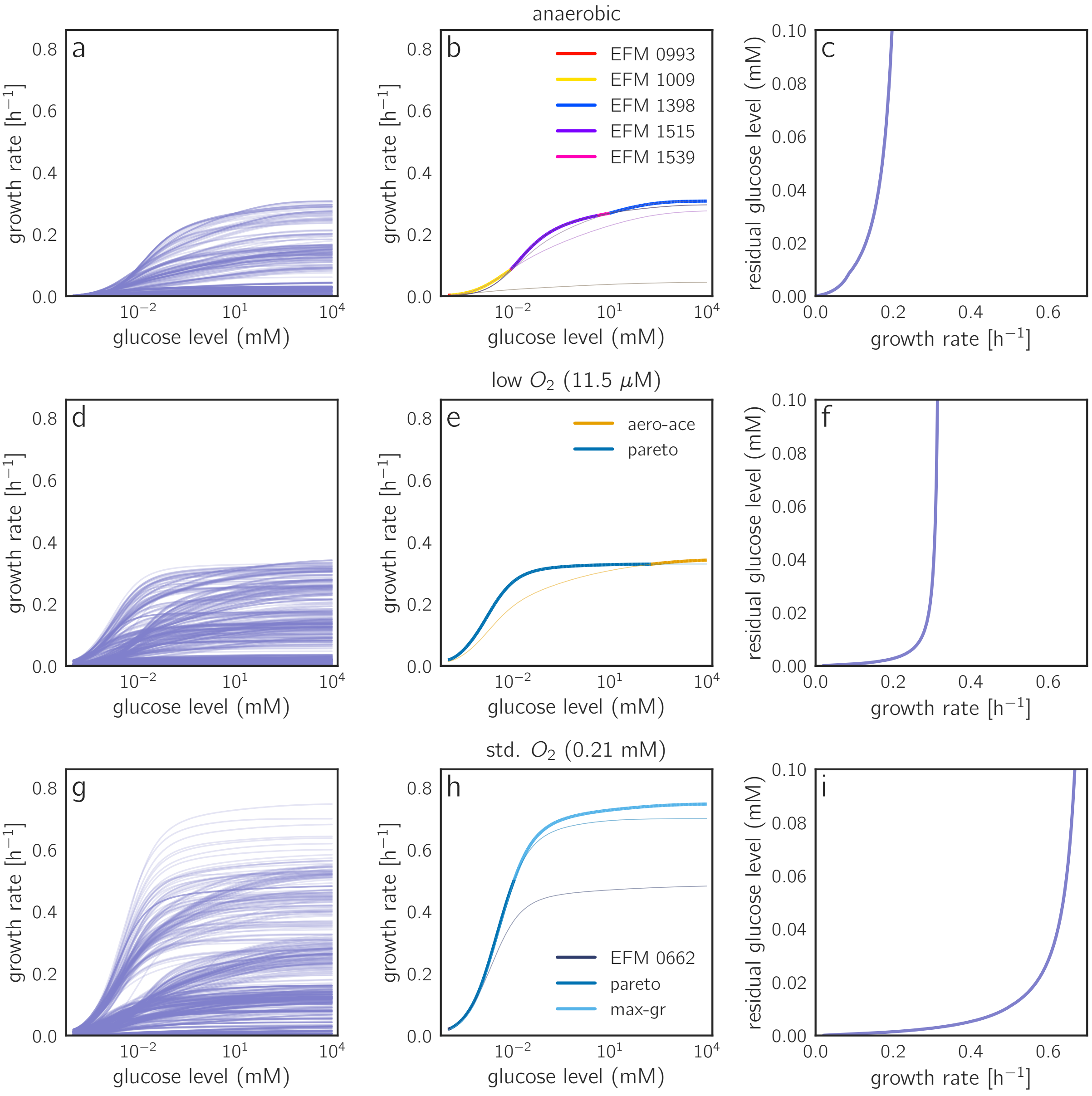

Figure S15: **Monod curves and residual glucose levels in chemostats.** All panels show predicted growth rates as functions of the external glucose level. The graphics in the first row refer to anaerobic conditions (vanishing oxygen level). The graphics in the other two rows show results for simulations with low (11.5 μM) and standard (0.21 mM) oxygen levels. (a) Growth rates of all anaerobic EFMs as functions of external glucose levels (curves extrapolated using a cubic function, 80 simulated points). (b) Highlighting the 5 EFMs that are optimal in at least one glucose concentration. The thick line follows the growth rate of the winning EFM and represents a predicted, kinetically justified Monod curve. (c) Predicted residual glucose level in chemostats as a function of cell growth rate. The curve is obtained from the thick curve in (b) by swapping the axes. Note that the glucose level is now shown on non-logarithmic scale, which changes the curve shape.

can be studied in combination. (ii) Our model predicts biomass yield and cell growth rate (instead of ATP yield and enzyme cost in glycolysis) as output variables. (iii) We screen the behavior for various glucose and oxygen levels, each providing different enzyme costs and growth-yield trade-offs. To assess the utility of EMP glycolysis, ED glycolysis, and respiration, we studied model variants in which these pathways were individually blocked. If a pathway is blocked, it may still be by-passed by other pathways, but if these pathways are less efficient the growth rate decreases. The quantitative decrease may depend, again, on the (glucose and oxygen) conditions. Here we compare our simulated wild-type *E. coli* (capable of using EMP

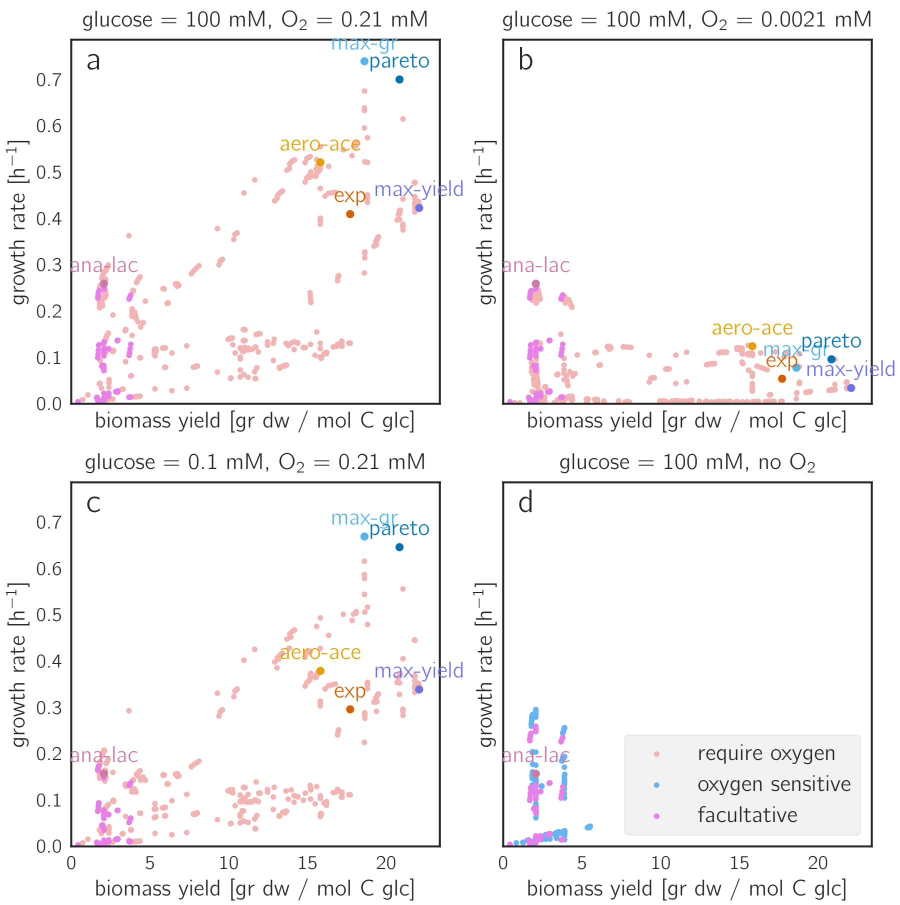

Figure S16: **Rate/yield trade-offs under different glucose and oxygen conditions.** The scatter plots show growth rate and biomass yield of all EFMs under standard conditions (a), low oxygen conditions (b), low glucose conditions (c), no oxygen (d). Lower glucose and oxygen levels lead to lower growth rates in many of the EFMs. Other EFMs may become optimal, and a wide Pareto front consisting of diverse EFMs may emerge, such as in panel (b).

glycolysis, ED glycolysis, and respiration) to variants in which one of the three pathways was blocked by a simulated gene deletion (see Figure 6 in main article).

Blocking either the EMP or ED pathway had the most marked effects at low oxygen levels, where respiration is not used and cells rely entirely on glycolysis for their ATP production. Blocking respiration had almost the same effect as setting the external oxygen concentration to very low values (except for the case of extremly low glucose concentrations). This analysis does not require any additional optimization runs. We just need to analyse the existing simulation results while discarding some EFMs, i.e. those that contain a blocked pathway. Note that our three model variants can also be seen as simple models of bacterial species that lack the genes for EMP glycolysis, ED glycolysis, or respiration.

In [30], it was hypothesized that non-respiring cells, which completely depend on ATP generated in glycolysis, should employ the high-yield EMP pathway despite it higher enzyme cost. Here we find the opposite: at (low oxygen and) high glucose levels, EMP and ED pathways yield approximately the same growth rates, and at low-to-medium glucose levels, the ED pathway performs even better, possibly due to ATP cost/yield ratio, which is still better than the cost/yield ratio of the EMP pathway – a conclusion that is in line with the simulation results, but not with the verbal conclusions, in [30].

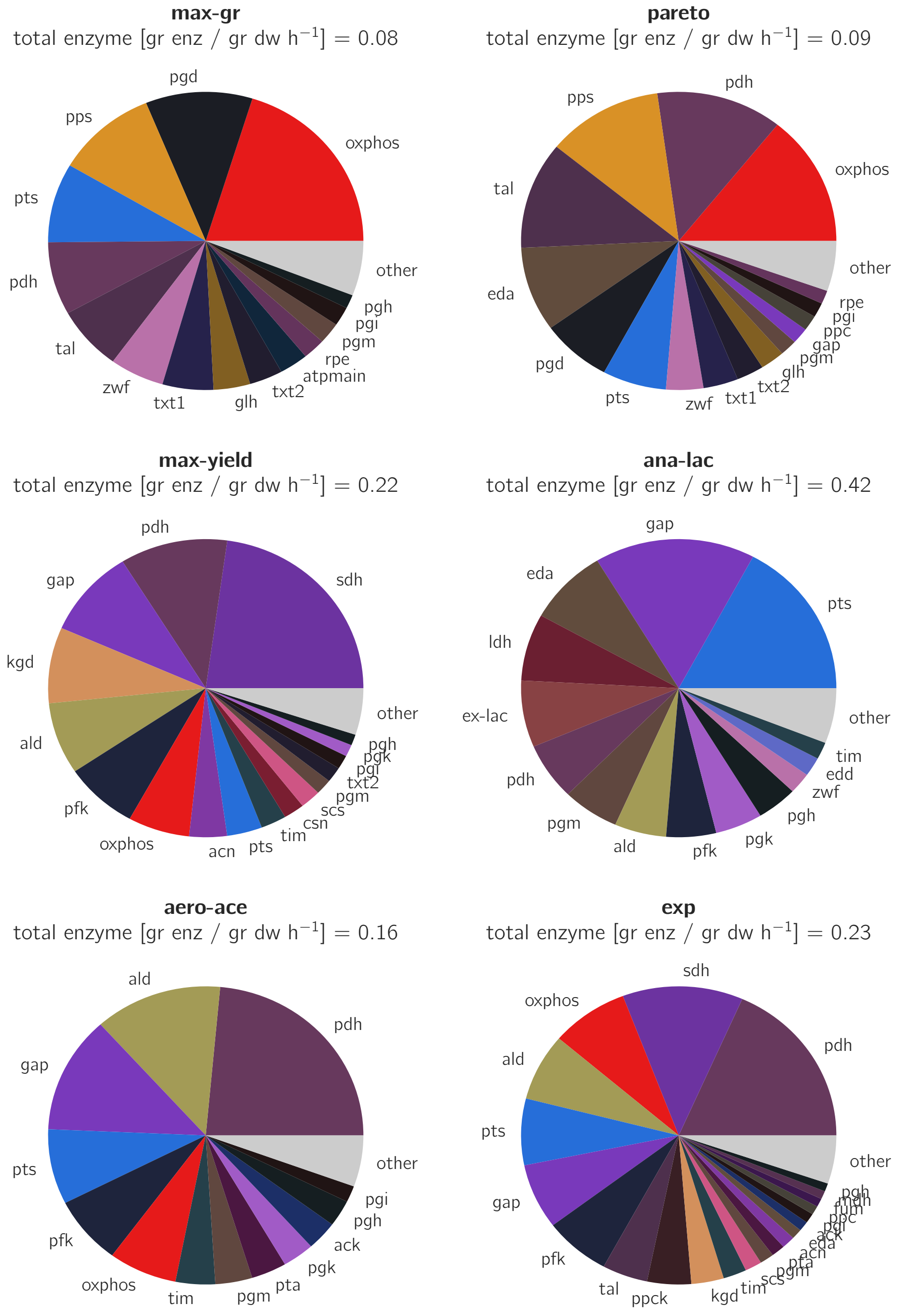

Figure S17: **Relative protein costs for selected EFMs (in standard conditions).** The reactions with the highest enzyme demand are: oxidative phosphorylation (**R80**), citrate synthase (**R21**), glucose uptake PTS system (**R1**) and glyceraldehyde-3P dehydrogenase (**R7ra**).

3.5 Epistatic effects between enzyme knockouts

Once growth rates and yields have been computed for all EFMs, simulating single, double, or multiple gene knockouts is really easy – we just need to exclude all EFMs that are affected by a knockout and redo the statistical analysis (e.g. finding the growth-maximising EFM, or determining the Pareto front). By comparing

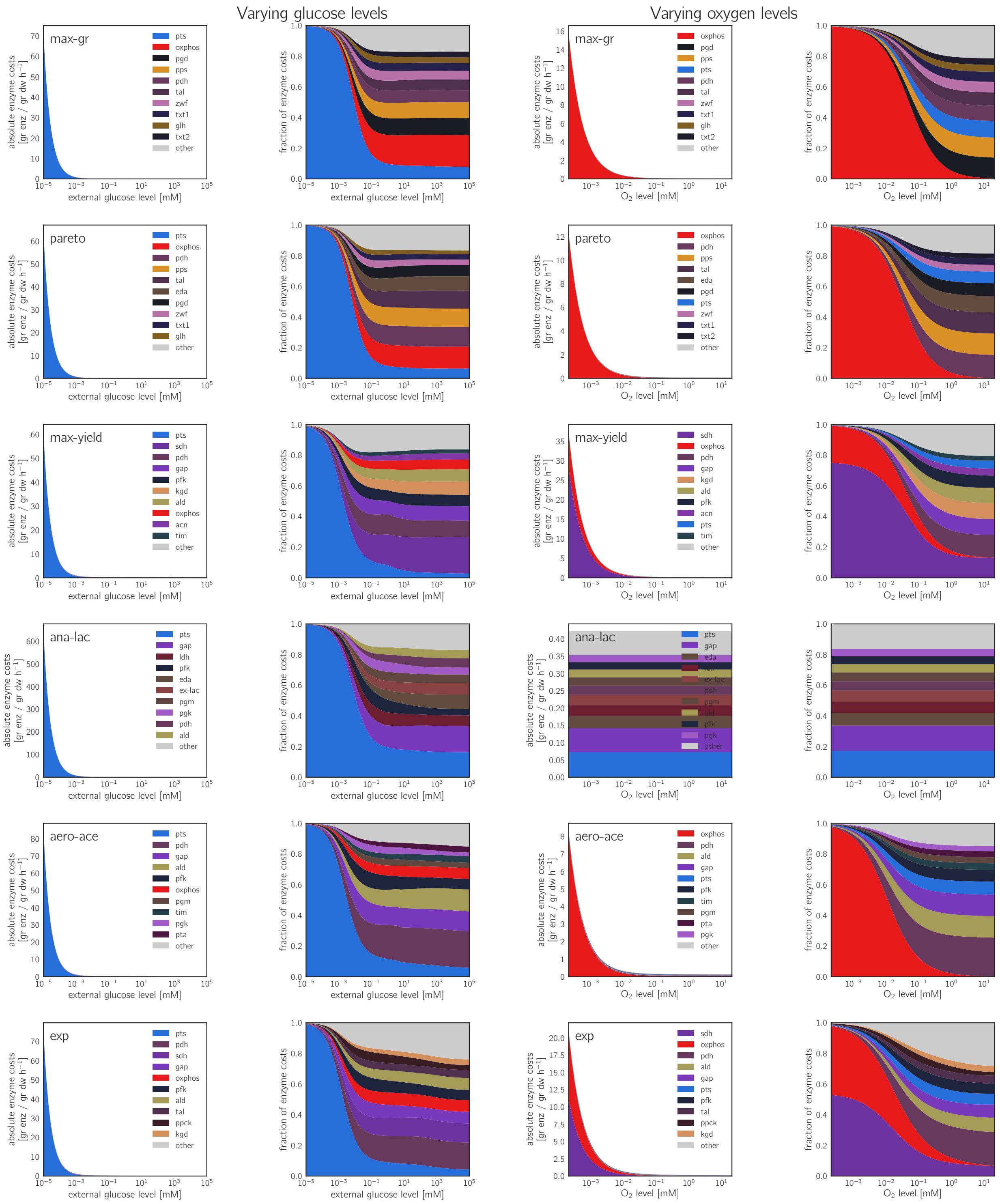

Figure S18: **Protein cost depending on varying glucose and oxygen levels.** The wto left columns show enzyme cost in absolute concentration (left) or as a fraction of the total cost (right) at varying glucose concentrations. Enzymes that require more than 5% of the total cost are shown in color; the enzyme cost of all other reactions, as a sum, is shown in the grey area denoted “other”. Only the six focal EFMs are shown. The two right columns show enzyme costs for varying oxygen concentration.

the performance of simulated single and double knockout strains, we can compute epistasis values. Epistasis describes whether a double knockout has a less severe or more severe effect than we would have expected based on the separate single knockouts, and can be defined based on growth rate or yield as the fitness objective.

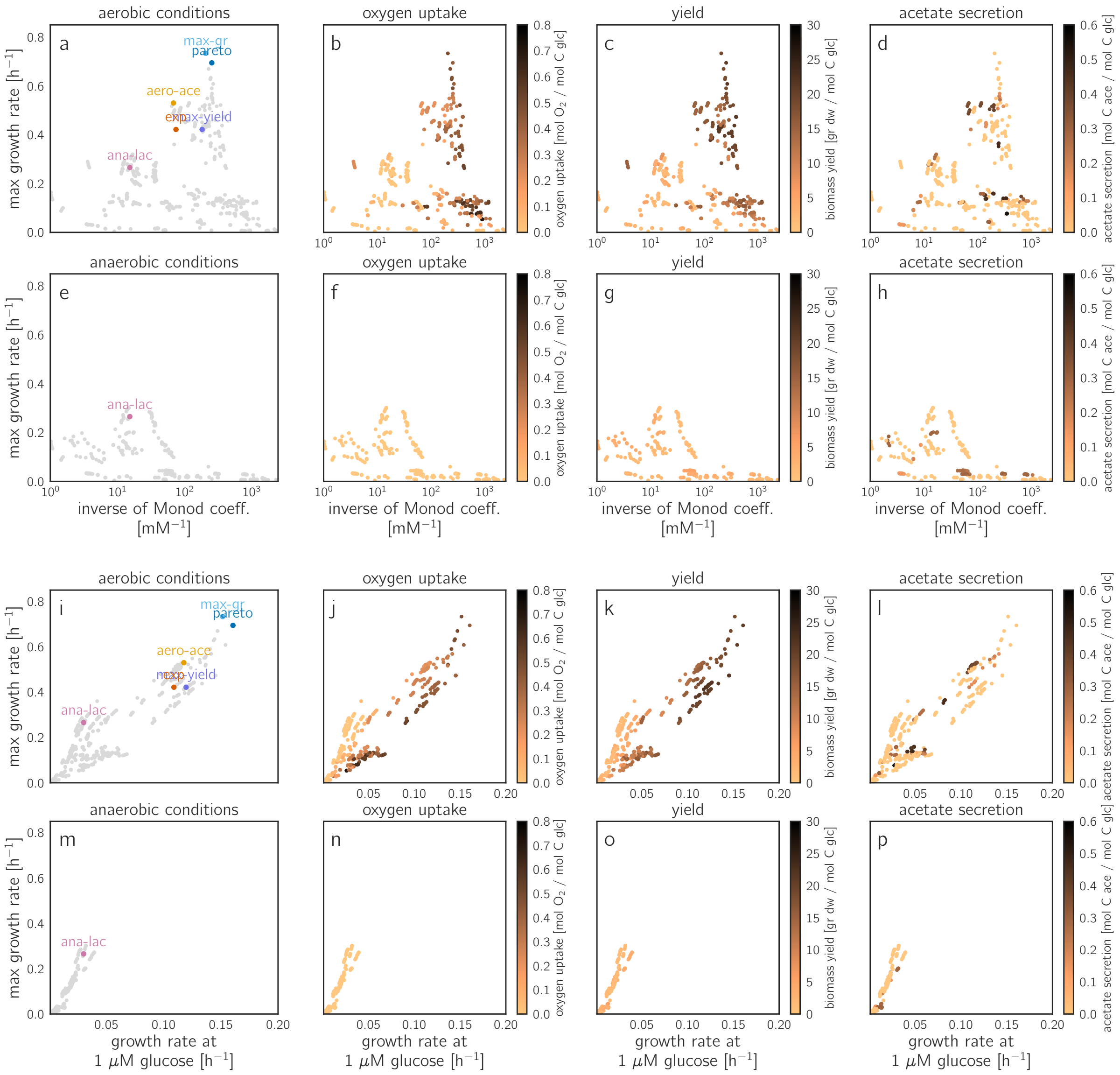

Figure S19: **Growth rates under high-glucose or low-glucose conditions.** The upper two rows (panels (a)-(h)) show scatter plots between the maximal growth rate at standard conditions (y-axis) and the inverse Monod coefficient. Likewise, the lower two rows (panels (i)-(p)) show scatter plots between the maximal growth rate at standard conditions (y-axis) and at very low glucose levels (1 μM). Odd rows show the distribution of all EFMs in aerobic conditions ($[O_2] = 0.21$ mM) and even rows show anaerobic conditions (only EFMs that do not consume O_2 are shown). The first column (left) displays all EFMs, and the three other columns show oxygen uptake, biomass yield and acetate secretion rate, respectively. In aerobic conditions there is almost no trade-off between the maximal growth rates at ligh or low glucose concentrations. This means that high-glucose conditions and low-glucose conditions often favor the same EFMs.

Figure S22 shows predicted epistasis values for growth rate as the selection objective. We find the same lethal knockouts as the yield (compare Figure S23). However, for growth rate there are fewer cases of positive epistatic interactions. After a knockout in lower glycosis (**R7**-**R8**), a second knockout in the pentose phosphate pathway (PPP, **R11**-**R15**) gives an extra reduction in growth rate (no positive scaled epistasis), while it does not have a big effect on the yield. There is a positive epistasis in the growth rate for the two reactions in the ED pathway (**R60** and **R61r**), while this pathway is not used in high-yield pathways (and therefore no epistasis there). Some of the epistatic interactions for yield reappear at low oxygen levels, because high-yield pathways tend to provide high growth rates at low oxygen levels (panel (d)). It is interesting that some epistatic interactions change signs between different conditions, such as the positive

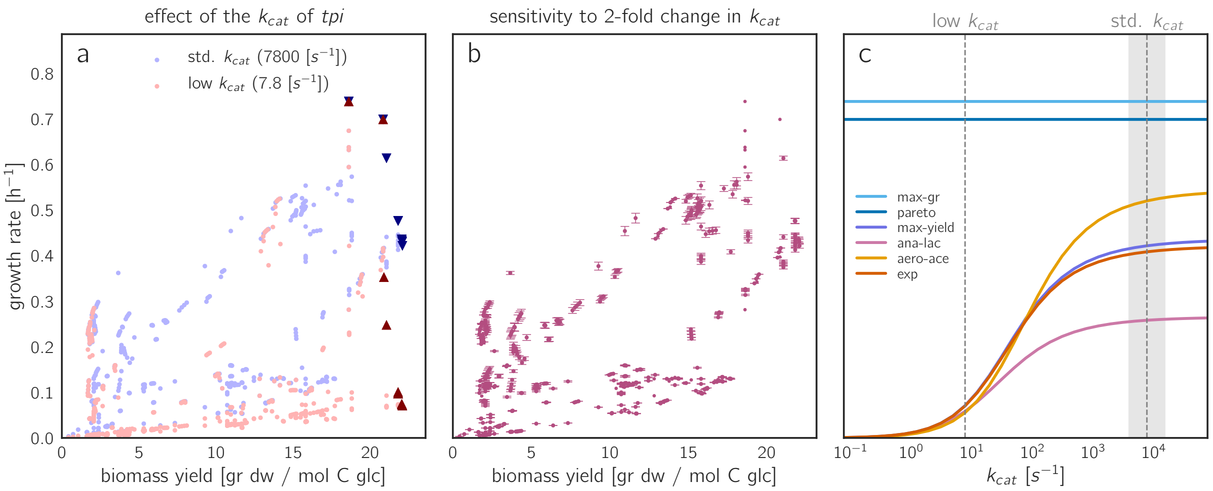

Figure S20: **Effects of a varying enzyme parameter on growth.** (a) Change in the rate/yield spectrum. We decreased the catalytic constant of triose-phosphate isomerase (*tpi*) by a factor of 1000 (from its original value 7800 s^{-1} to 7.8 s^{-1}). (b) Sensitivity of the growth rate to the k_{cat} value of *tpi* for all different EFMs. Error bars depict the change in growth rate caused by a 50% increase or decrease in the k_{cat} value. To compute the sensitivities, we used a formula that assumes a direct compensation of the affected enzyme (see SI section 4.3). (c) Effect of a variable k_{cat} value of *tpi* (x-axis) on the growth rates of selected EFMs. The 50% range around the standard k_{cat} value is marked by grey shading. The top two EFMs *max-gr* and *pareto* do not use *tpi* at all and are thus insensitive to changes in its k_{cat}.

epistasis between uptake (**R1**) with the ED pathway (**R60** and **R61r**) (panels (b) and (f)) which turns negative at low oxygen levels (panel (d)).

The epistatic effects on yield are shown in Figure S23. Panel (a) shows the relative yields of double knockouts as a fraction of the maximal yield (i.e. the yield of the wild-type). Single knockouts are shown on the diagonal. Obviously, the uptake reaction (**R1**) and biomass reaction (**R70**) are essential. As can be seen, a knockout of reactions **R21-R24** and **R40** is lethal because there is no way to produce 2-oxogluterate, which is needed for the biomass reaction (although this is not directly obvious for reaction **R20** and **R40**). Other essential reactions are **R93** (to obtain ammonia) and **R12r** (to obtain ribose-5-P). Single knockouts in oxidative phosphorylation (**R80**) or lower glycolysis (**R7**/**R8**) decrease the yield, because those are used by the high-yield EFMs. Some double knockouts are synthetically lethal—i.e. only the double knockout is lethal but each of the single knockouts is viable. These are mostly combinations of knockouts in lower glycolysis, the ED-pathway and the pentose phosphate pathway. A few double knockouts lower the yield dramatically, mostly combinations of the PPP and the TCA cycle. In panel (b), the scaled yield epistatis is calculated with the formula

$$\frac{Y_{1,2} - Y_1 \cdot Y_2}{|\widetilde{Y}_{1,2} - Y_1 \cdot Y_2|}\,, \tag{19}$$

where Y_i is the scaled yield of knockout i and $\widetilde{Y}_{1,2} = \min(Y_1, Y_2)$ if $\widetilde{Y}_{1,2} > Y_1 \cdot Y_2$ and $\widetilde{Y}_{1,2} = 0$ otherwise (definition from [31]). Here we can clearly see the synthetically lethal double knockouts and the double knockouts that have a dramatically lower yield in red. In blue (i.e. epistasis score = 1) we find combinations or knockouts where one is dominant, while the other one doesn't reduce the yield any further. For instance, this is the case for sequential reactions in the same pathway (e.g. **R10a** and **R10b**).

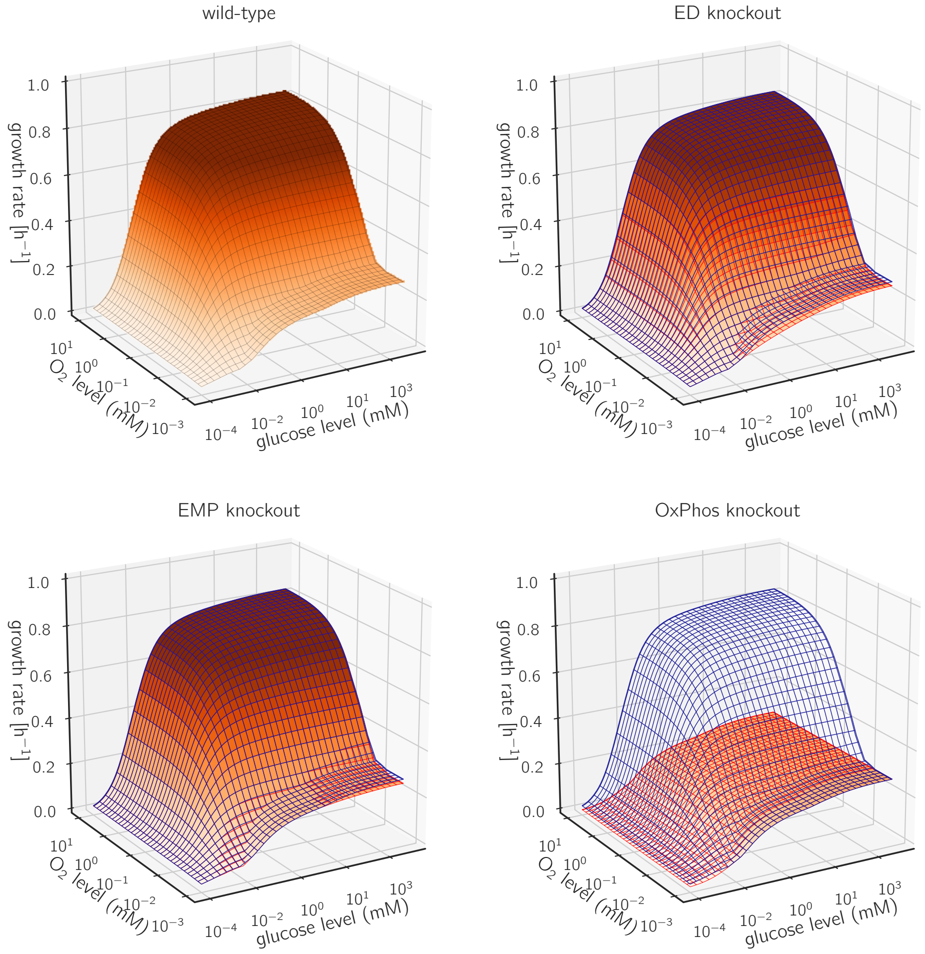

Figure S21: **Monod surface plots for strains deficient in EMP glycolysis, ED glycolysis, or respiration.** (a) Predicted growth rates, as a function of glucose and oxygen level, for simulated wild-type *E. coli*. (b) Changes in growth after a simulated suppression of the ED pathway (wild-type surface shown in blue, mutant surface shown in red). (c) Changes in growth after a simulated suppression of EMP pathway. (d) Changes in growth after a simulated suppression of respiration. Compare Figure 6 in the main article, which is based on the same data.

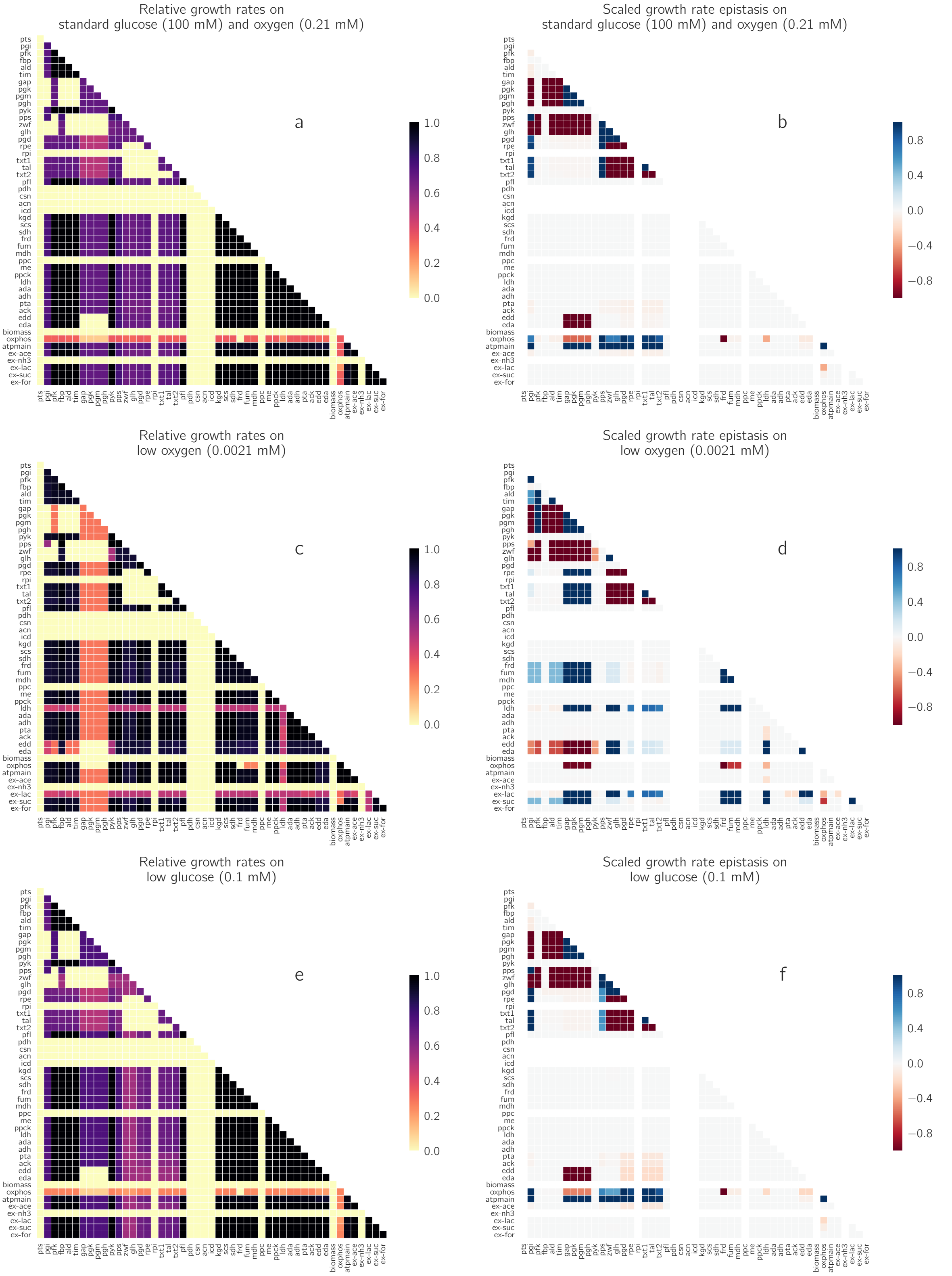

Figure S22: **Epistatis effect of double knockouts on growth rate.** Relative growth rate (a,c,e) and scaled growth rate epistasis (b,d,f). Epistasis values are computed using Equation (19), but based on growth rates instead of biomass yields. The three rows refer to standard conditions (a-b), low oxygen conditions (c-d), and low glucose conditions (e-f) respectively. The relative growth rates of single reaction knockouts are shown on the diagonal (only in the left column).

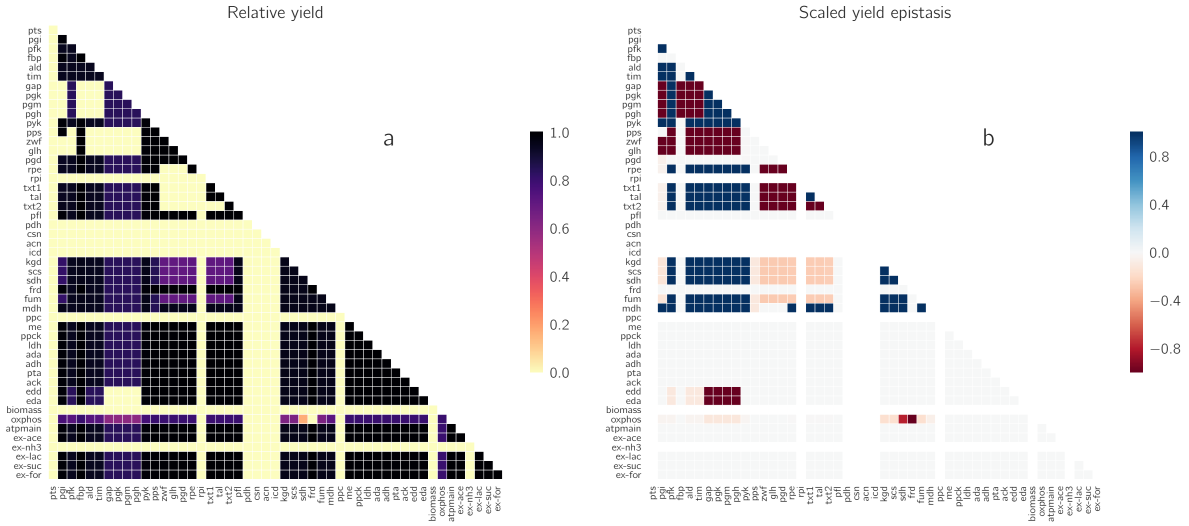

Figure S23: **Epistatic effect of double knockouts on biomass yield.** (a) Relative yields of double knockouts as a fraction of the maximal yield (i.e. the yield of the wild-type). (b) Scaled epistatis for biomass yields.

4 Mathematical derivations

4.1 Computing a stationary flux distribution in a metabolic network from measured fluxes

Assume that absolute fluxes have been measured using ^{13}C metabolic flux analysis (MFA), but the list of fluxes does not cover all the 52 reactions in our CCM model. We now describe a method to estimate the missing reaction fluxes (and maybe adjust the measured ones) to obtain a consistent flux distribution, i.e. one that satisfies the constrains of the system, including mass balance. One option is to find the flux mode (v) that minimizes the l_1 distance to the measured exchange fluxes ($\bar{v} \pm \sigma$) and fulfills mass balance constraints:

$$\begin{aligned} \text{minimize} \quad & \sum_{i \in I_{\text{meas}}} |v_i - \hat{v}_i| \\ Sv &= 0 \\ v_{min} \leq & \; v \leq v_{max} \\ \bar{v}_i - \sigma_i \leq & \; \hat{v}_i \leq \bar{v}_i + \sigma_i, \end{aligned} \tag{20}$$

where $\hat{v}$ is an auxiliary variable that represents the actual flux, constrained to be within the confidence interval of the absolute measured fluxes. This linear programming problem may have non-unique solutions. To reduce the set of solutions, we add a secondary optimization goal, namely to maximize ATP production (i.e. in our model, to maximize the reaction flux v_{r82}).

$$\begin{aligned} \text{maximize} \quad & v_{r82} \\ \sum_{i \in I_{\text{meas}}} |v_i - \hat{v}_i| &= \epsilon_1 \\ Sv &= 0 \\ v_{min} \leq & \; v \leq v_{max} \\ \bar{v}_i - \sigma_i \leq & \; \hat{v}_i \leq \bar{v}_i + \sigma_i, \end{aligned} \tag{21}$$

where ϵ_1 is the minimum value achieved in the first optimization.

4.2 Global cost sensitivities can be approximated by local cost sensitivities

We now compute the sensitivities between enzyme cost and model parameters such as k_{cat} values. In analogy to the local and global flux sensitivities in Metabolic Control Analysis, called elasticities and flux response coefficients, we distinguish local and global sensitivities for enzyme cost at predefined fluxes. From these sensitivities, we can compute local and global sensitivities between parameter perturbations and the cell growth rate. To define local sensitivities, we perturb a parameter and adapt only the enzyme of the affected reaction (while all metabolite levels and fluxes must remain unchanged). To define the global sensitivities, we perturb the same parameter and adapt all enzymes (and, accordingly, all metabolite levels (where the flux must remain the same and the cost-optimal adaptation is chosen). Below we show that, for small parameter changes, local and global adaptation lead to the same first-order cost changes, i.e. that local and global sensitivities are identical.

To define the optimal enzyme cost $E_{\text{met}}^{\text{opt}}$ and the optimal logarithmic metabolite profile s_0 for a given flux

vector v, we solve the enzyme cost minimization problem

$$\begin{aligned} E_{\rm met}^{\rm opt}(v,k) &= \min_s E_{\rm met}(v,s,k) \\ s^{\rm opt}(v,k) &= \text{argmin}_s E_{\rm met}(v,s,k) \end{aligned} \tag{22}$$

with an enzymatic cost function $E_{\rm met}(v,s,k)$ derived from a kinetic model. The fluxes v are fixed and given, the vector of logarithmic metabolite levels s can be varied within the metabolite polytope, and the vector k contains model parameters that affect individual reaction rates.

To show that local and global sensitivities are identical, we start from the unperturbed reference values k_0 and the resulting optimal metabolite vector s_0; then we expand the enzymatic cost function quadratically around this point with respect to s. To simplify the notation (and without loss of generality), we consider a one-dimensional problem (with scalar logarithmic concentration s):

$$E_{\rm met}(v,s,k) \approx a(s-s_0)^2 + \underbrace{b}_{0}(s-s_0) + c, \tag{23}$$

with expansion coefficients a, b, and c. Since we expand around an optimum point, the coefficient b vanishes. Now we consider a small parameter change $\mathrm{d}k$, which changes the cost landscape (and thereby the expansion coefficients):

$$E_{\rm met}(v,s,k+\mathrm{d}k) \approx [a+\alpha\,\mathrm{d}k]\,(s-s_0)^2 + [\beta\,\mathrm{d}k]\,(s-s_0) + [c+\gamma\,\mathrm{d}k]. \tag{24}$$

We compute the new optimum point $s^{\rm opt*}$ by equating the derivative to zero:

$$\begin{aligned} 0 &= 2[a+\alpha\,\mathrm{d}k]\,(s^{\rm opt*}-s_0) + [\beta\,\mathrm{d}k] \\ \Rightarrow s^{\rm opt*} &= s_0 - \frac{\beta\,\mathrm{d}k}{2(a+\alpha\,\mathrm{d}k)} \approx s_0 - \frac{\beta\,\mathrm{d}k}{2\,a}, \end{aligned} \tag{25}$$

where we assume that $a \geq 0$ (i.e. we assume that we started from a unique optimum state). To compute the resulting cost, we insert $s^{\rm opt*}$ back into the perturbed cost function:

$$\begin{aligned} E_{\rm met}^{\rm opt}(v,k+\mathrm{d}k) &= E_{\rm met}(v,s^{\rm opt*}(k+\mathrm{d}k),k+\mathrm{d}k) \\ &\approx \underbrace{[a+\alpha\,\mathrm{d}k](-\frac{\beta\,\mathrm{d}k}{2\,a})^2 + [\beta\,\mathrm{d}k]\,(-\frac{\beta\,\mathrm{d}k}{2\,a})}_{\text{second-order terms in } \mathrm{d}k} + \underbrace{[c+\gamma\,\mathrm{d}k]}_{\approx E_{\rm met}(v,s_0,k+\mathrm{d}k)}. \end{aligned} \tag{26}$$

This means:

$$\frac{\partial E_{\rm met}^{\rm opt}(v,k)}{\partial k} = \frac{\partial E_{\rm met}(v,s^{\rm opt}(k),k)}{\partial k} = \frac{\partial E_{\rm met}(v,s,k)}{\partial k}|_{s=s_0}. \tag{27}$$

In other words: the first-order cost change after a parameter perturbation, and with an optimal adaptation of all enzyme levels, is given by the cost change that would ensue from adapting only the affected enzyme (and keeping all metabolite levels unchanged). Note that our first-order expansion holds only for small (relative) parameter changes.

4.3 Enzyme cost sensitivities of kinetic constants

Cost sensitivities for $k_{\rm cat}$ values In the case of $k_{\rm cat}$ values, we obtain a simple formula for the local cost sensitivities: with the rate law $v = E\ k\ f(s)$, with fixed flux v and logarithmic metabolite levels s (and thus fixed f), we obtain $E_{\rm adapt} \cdot k_{\rm adapt} = E_{\rm ref} \cdot k_{\rm ref} = \frac{v}{f}$ = const. (where "ref" denotes the reference state, and

"adapt" the perturbed state with adapted enzyme level). The adapted enzyme level reads $E_{\rm adapt} = E_{\rm ref}\,\frac{k_{\rm ref}}{k_{\rm adapt}}$ and its derivative reads $\frac{\partial E_{\rm adapt}}{\partial k_{\rm adapt}} = -\frac{E_{\rm ref}\;k_{\rm ref}}{k^2_{\rm adapt}}$. In the original reference state (where $k_{\rm adapt} = k_{\rm ref}$) we obtain

$$\frac{\partial E_{\rm adapt}}{\partial k_{\rm adapt}} = -\frac{E_{\rm ref}}{k_{\rm ref}}.$$

The local cost sensitivity (with enzyme cost weight, i.e. molecular mass w) thus reads

$$\frac{\partial E_{\rm met}}{\partial k} = -w\,\frac{E_{\rm ref}}{k_{\rm ref}} = -\frac{E^{\rm ref}_{\rm met}}{k_{\rm ref}}.$$

As we saw before, these (first-order) local cost sensitivities are identical to the (first-order) global cost sensitivities.

Cost sensitivities for $K_{\rm eq}$ values To compute the cost sensitivity of an independent change in one of the $K_{\rm eq}$ values, we first calculate the elasticity

$$\begin{aligned} E_{\rm met} &= w\,v\cdot k_{\rm cat}^{-1}\cdot \eta^{\rm sat}(\mathbf{c})^{-1}\cdot\left[1 - Q(\mathbf{c})K_{\rm eq}^{-1}\right]^{-1} \\ \frac{\partial E_{\rm met}}{\partial K_{\rm eq}} &= \frac{E_{\rm met}}{K_{\rm eq}}\cdot\frac{\partial \log E_{\rm met}}{\partial \log K_{\rm eq}} = -\frac{E_{\rm met}}{K_{\rm eq}}\cdot\frac{QK_{\rm eq}^{-1}}{1-QK_{\rm eq}^{-1}} \\ &= -\frac{E_{\rm met}}{K_{\rm eq}}\cdot\frac{1}{K_{\rm eq}Q^{-1}-1}, \end{aligned} \tag{28}$$

where $Q(\mathbf{c})$ is the mass-action ratio, and $\eta^{\rm sat}(\mathbf{c})$ represents the saturation efficiency (which does not depend on $K_{\rm eq}$). Using the definition $\eta^{\rm thr} = \left(1 - Q(\mathbf{c})K_{\rm eq}^{-1}\right)$, we can rewrite this result as

$$\frac{\partial E_{\rm met}}{\partial K_{\rm eq}} = -\frac{E_{\rm met}}{K_{\rm eq}}\cdot\left[\eta^{\rm thr}(\mathbf{c})^{-1} - 1\right]. \tag{29}$$

Note that this formula refers to individual changes in $K_{\rm eq}$ values; it does not account for dependencies between $K_{\rm eq}$ values due to Wegscheider conditions.

Cost sensitivities for K_M values (reaction substrates) The expression for the cost sensitivity of a K_M value is given by

$$\begin{aligned} E_{\rm met} &= w\;v\;k_{\rm cat}^{-1}\;\eta^{\rm thr}(\mathbf{c})^{-1}\cdot\left[\frac{\prod_j \frac{c_j}{K_{M,c_j}}}{\prod_j\left(1+\frac{c_j}{K_{M,c_j}}\right)+\prod_k\left(1+\frac{c_k}{K_{M,c_k}}\right)-1}\right]^{-1} \\ &= w\;v\;k_{\rm cat}^{-1}\;\eta^{\rm thr}(\mathbf{c})^{-1}\cdot\left[\prod_j\left(\frac{K_{M,c_j}}{c_j}+1\right)+\prod_j\left(\frac{K_{M,c_j}}{c_j}\right)\cdot\left(\prod_k\left(1+\frac{c_k}{K_{M,c_k}}\right)-1\right)\right], \end{aligned} \tag{30}$$

where j is the index for $\mathbf{c}$ for all substrates and k for all products. We will calculate the sensitivity to changes in the Michaelis-Menten constant of a substrate s, which we denote by $K_s \equiv K_{M,c_s}$. For this cost function,

the K_M sensitivity would be

$$
\begin{aligned}
\frac{\partial E_{\text{met}}}{\partial K_s} &= w\ v\ k_{\text{cat}}^{-1}\ \eta^{\text{thr}}(\mathbf{c})^{-1} \cdot \left[\frac{1}{c_s} \prod_{j \neq s} \left(\frac{K_{M,c_j}}{c_j} + 1 \right) + \frac{1}{c_s} \prod_{j \neq s} \frac{K_{M,c_j}}{c_j} \cdot \left(\prod_k \left(1 + \frac{c_k}{K_{M,c_k}} \right) - 1 \right) \right] \\
&= \frac{E_{\text{met}}\ \eta^{\text{sat}}(\mathbf{c})}{K_s} \left[\frac{K_s}{c_s} \prod_{j \neq s} \left(\frac{K_{M,c_j}}{c_j} + 1 \right) + \frac{K_s}{c_s} \prod_{j \neq s} \frac{K_{M,c_j}}{c_j} \cdot \left(\prod_k \left(1 + \frac{c_k}{K_{M,c_k}} \right) - 1 \right) \right] \\
&= \frac{E_{\text{met}}\ \eta^{\text{sat}}(\mathbf{c})}{K_s} \left[\prod_j \left(\frac{K_{M,c_j}}{c_j} + 1 \right) - \prod_{j \neq s} \left(\frac{K_{M,c_j}}{c_j} + 1 \right) + \prod_j \frac{K_{M,c_j}}{c_j} \cdot \left(\prod_k \left(1 + \frac{c_k}{K_{M,c_k}} \right) - 1 \right) \right] \\
&= \frac{E_{\text{met}}\ \eta^{\text{sat}}(\mathbf{c})}{K_s} \left[\eta^{\text{sat}}(\mathbf{c})^{-1} - \prod_{j \neq s} \left(\frac{K_{M,c_j}}{c_j} + 1 \right) \right] \\
&= \frac{E_{\text{met}}}{K_s} \left[1 - \eta^{\text{sat}}(\mathbf{c}) \prod_{j \neq s} \left(\frac{K_{M,c_j}}{c_j} + 1 \right) \right]. \qquad (31)
\end{aligned}
$$

If s is the only substrate for this reaction, we are left with

$$\frac{\partial E_{\text{met}}}{\partial K_s} = \frac{E_{\text{met}}}{K_s} \left[1 - \eta^{\text{sat}}(\mathbf{c}) \right]. \qquad (32)$$

Again, this formula refers to individual changes in K_{M} values; it does not account for dependencies due to Haldane relationships.

Cost sensitivities for K_M values (reaction products) We start again with equation (30) and take the derivative with respect to $K_p \equiv K_{M,c_p}$ – the Michaelis-Menten constant to one of the products. In this case, the sensitivity is given by

$$
\begin{aligned}
\frac{\partial q}{\partial K_p} &= -\frac{E_{\text{met}}}{K_p} \cdot \eta^{\text{sat}}(\mathbf{c}) \cdot \prod_j \frac{K_{M,c_j}}{c_j} \cdot \prod_k \frac{c_k}{K_{M,c_k}} \cdot \prod_{k \neq p} \left(\frac{K_{M,c_k}}{c_k} + 1 \right) \\
&= -\frac{E_{\text{met}}}{K_p} \cdot \eta^{\text{sat}}(\mathbf{c}) \cdot Q(\mathbf{c}) \cdot \frac{\prod_j K_{M,c_j}}{\prod_k K_{M,c_k}} \cdot \prod_{k \neq p} \left(\frac{K_{M,c_k}}{c_k} + 1 \right), \qquad (33)
\end{aligned}
$$

and in case there is only one substrate and one product, this simplifies to

$$\frac{\partial E_{\text{met}}}{\partial K_p} = -\frac{E_{\text{met}}}{K_p} \cdot \frac{\prod_k \frac{c_k}{K_{M,c_k}}}{1 + \prod_k \frac{c_j}{K_{M,c_j}} + \prod_k \frac{c_k}{K_{M,c_k}}}. \qquad (34)$$

4.4 Growth-rate sensitivities of kinetic constants

Since we use a nonlinear function to convert enzyme cost (E_{met}) into growth rate (μ), the sensitivities of μ to local changes in a kinetic constant (k) require an additional prefactor, i.e. according to the chain rule:

$$\frac{\partial \mu}{\partial k} = \frac{\partial \mu}{\partial E_{\text{met}}} \cdot \frac{\partial E_{\text{met}}}{\partial k}. \qquad (35)$$

For the second term on the right, one can use the results from the previous section on cost sensitivities (SI section 4.3), replacing k with either k_{cat}, K_s, K_p, or K_{eq}. The first term on the right does not depend on the type of kinetic constant, and can be expressed as a function of μ. To compute it, we must first express μ as a

direct function of $E_{\rm met}$:

$$r_{\rm BM} = \frac{v_{\rm BM}}{E_{\rm met}} = \frac{v_{\rm BM}}{E_{\rm met}} \quad (36)$$

$$\mu = \frac{f_{\rm prot}\; a\; r_{\rm BM}}{1 + b\; r_{\rm BM}\; f_{\rm prot}} = \frac{f_{\rm prot}\; a\; v_{\rm BM}}{E_{\rm met} + b\; v_{\rm BM}\; f_{\rm prot}}. \quad (37)$$

Therefore, the derivative would be:

$$\frac{\partial \mu}{\partial E_{\rm met}} = -\frac{f_{\rm prot}\; a\; v_{\rm BM}}{(E_{\rm met} + b\; v_{\rm BM}\; f_{\rm prot})^2} = -\frac{\mu^2}{f_{\rm prot}\; a\; v_{\rm BM}}. \quad (38)$$

Quite often, it is useful to calculate the scaled sensitivity:

$$\frac{\partial \ln \mu}{\partial \ln E_{\rm met}} = -\frac{E_{\rm met}}{E_{\rm met} + b\; v_{\rm BM}\; f_{\rm prot}} = \frac{b}{a}\; \mu - 1. \quad (39)$$

If a kinetic parameter changes from a reference value k_0 to a value k, we can use the first-order Taylor expansion for μ in log-scale:

$$\begin{aligned}
\ln \mu &= \ln \mu_0 + \left.\frac{\partial \ln \mu}{\partial \ln k}\right|_{k_0} (\ln k - \ln k_0) + \ldots \\
\ln\left(\frac{\mu}{\mu_0}\right) &\approx \ln\left(\frac{k}{k_0}\right) \cdot \left.\frac{\partial \ln \mu}{\partial \ln k}\right|_{k_0} = \ln\left(\frac{k}{k_0}\right) \cdot \left.\frac{\partial \ln \mu}{\partial \ln E_{\rm met}}\right|_{k_0} \cdot \left.\frac{\partial \ln E_{\rm met}}{\partial \ln k}\right|_{k_0} \\
&= \ln\left(\frac{k}{k_0}\right) \cdot \left(\frac{b}{a}\; \mu_0 - 1\right) \cdot \left.\frac{\partial \ln E_{\rm met}}{\partial \ln k}\right|_{k_0} \quad (40)
\end{aligned}$$

4.5 Increasing a $k_{\rm cat}$ value cannot increase the enzyme demand

Proposition 1 *A local increase in an enzyme's $k_{\rm cat}$ value can never increase the optimal total enzyme cost.*

Proof Let $E^{\rm opt}_{\rm met}(v, k) \equiv \min_s E_{\rm met}(v, s, k)$ be the minimal enzyme cost required (optimized over all metabolite level profiles s) for a given flux vector v and kinetic parameter set k. Let $\hat{k}$ be another kinetic parameters set which is identical to k except for the $k_{\rm cat}$ of a single enzyme i, specifically $\hat{k}_{{\rm cat},i} > k_{{\rm cat},i}$. Now, for any metabolite profile s, if we compare $E_{\rm met}(v, s, \hat{k})$ to $E_{\rm met}(v, s, k)$ the only change would be the cost associated with that one enzyme, so

$$\begin{aligned}
&E_{\rm met}(v, s, \hat{k}) - E_{\rm met}(v, s, k) = E_i(v_i, s, \hat{k}) - E_i(v_i, s, k) \\
&= \tfrac{h_i v_i}{\hat{k}_{{\rm cat},i}\; f(s)} - \tfrac{h_i v_i}{k_{{\rm cat},i}\; f(s)} = \tfrac{h_i v_i}{f(s)} \left(\hat{k}^{-1}_{{\rm cat},i} - k^{-1}_{{\rm cat},i}\right) \leq 0\;. \quad (41)
\end{aligned}$$

Therefore, $s \quad E_{\rm met}(v, s, \hat{k}) \leq E_{\rm met}(v, s, k)$ holds for all metabolute profiles s, and this inequality will also trivially apply to the minima of both functions.

4.6 Approximation formula for Monod curves and higher-dimensional Monod functions

A Monod function – the cellular growth rate as a function of extracellular metabolite concentrations – can have a complicated shape. However, we can obtain a simple formula with interpretable parameters based on the logic of EFCM and on some simplifying assumptions:

1. We assume that the cell is bound to use a single, predefined flux mode (e.g. an EFM), and compute the Monod surface for this flux mode.
2. We assume that the metabolic enzymes occupy a fixed fraction of the biomass.
3. As an approximation, we assume that all internal metabolite levels remain constant despite changes of the external nutrient levels; this implies that (given our flux mode) all internal enzyme levels are constant, too, and that only the transporter levels are changing – the same type of approximation that was made in satFBA [12].

The first assumption will later be dropped, and the second one can be avoided[3]. To derive our formula, we consider a kinetic model and split the total cost of metabolic enzymes into

$$E_{\mathrm{met}} = \underbrace{\sum_t w_t c_t^{\mathrm{trans}}}_{E_{\mathrm{transporter}}} + \underbrace{\sum_i w_i\, c_i^{\mathrm{enz}}}_{E_{\mathrm{internal}}}, \tag{42}$$

where w_t and w_i denote cost weights (e.g. protein masses) for transporters and metabolic enzymes, respectively. With the yields $y_t = \frac{v_{\mathrm{bm}}}{v_t}$ for the different substances taken up, the external substance concentrations x_t, and the transporter rate laws $v_t = c_t^{\mathrm{trans}} f_t(x_t)$, we can solve for the transporter concentrations $c_t^{\mathrm{trans}} = \frac{v_t}{f_t(x_t)} = \frac{v_{\mathrm{bm}}}{y_t\, f_t(x_t)}$ and write the enzyme cost as

$$E_{\mathrm{met}} = \sum_t \underbrace{\left(\frac{w_t}{y_t}\right)}_{\alpha_t} \frac{v_{\mathrm{bm}}}{f_t(x_t)} + \underbrace{\left(\frac{E_{\mathrm{internal}}}{v_{\mathrm{bm}}}\right)}_{\beta} v_{\mathrm{bm}}, \tag{43}$$

where the expressions in round brackets are constant for the given flux mode. From now on we call these expressions α_t and β for ease of notation. The α_t depend directly on the shape of the flux mode, while β depends also on kinetics and needs to be computed by ECM. In a full EFCM analysis, β would also depend on the external concentrations x_t, but based on Assumption 3, we treat it as constant, and compute it once for a typical choice of external concentrations. Now we can compute the cell growth rate:

$$\mu = \frac{v_{\mathrm{bm}}}{c_{\mathrm{bm}}} = \underbrace{\frac{E_{\mathrm{met}}}{c_{\mathrm{bm}}}}_{\gamma} \cdot \frac{v_{\mathrm{bm}}}{E_{\mathrm{met}}} = \gamma \cdot \frac{v_{\mathrm{bm}}}{\sum_t \alpha_t \frac{v_{\mathrm{bm}}}{f_t(x_t)} + \beta\, v_{\mathrm{bm}}} = \gamma \cdot \frac{1}{\sum_t \frac{\alpha_t}{f_t(x_t)} + \beta}, \tag{44}$$

where γ (the fraction of metabolic enzyme within the biomass) is assumed to be constant[4]. In analogy to Eq. (), we can express it as $\gamma = f_{\mathrm{ccm}}\, f_{\mathrm{prot}}$. Now we choose rate laws for the transporters. We first assume an irreversible mass-action rate law $f_t(x_t) = k_t\, x_t$ with a rate constant k_t. Inserting this into Eq. (44), we obtain

$$\mu = \gamma \cdot \frac{1}{\sum_t \frac{\alpha'_t}{x_t} + \beta}, \tag{45}$$

where $\alpha'_t = \alpha_t / k_t$. Alternatively, we may assume an irreversible Michaelis-Menten transporter kinetics $f_t(x_t) = \frac{k_t\, x_t}{K_t + x_t}$ with rate constant k_t and Michaelis-Menten constant K_t. Inserting this into Eq. (44), we

[3] We make this assumption just for ease of demonstration. A similar formula, based on a growth-rate-dependent enzyme fraction, could be derived as in section 1.3.

[4] As noted above, this assumption can be dropped. If we replace γ by a linear function $a + b\,\mu$ with constant coefficients, the equation can still be easily solved for μ (see the corresponding derivation in section 1.3). In the resulting formula, we obtain another saturable function around around the fraction.

obtain

$$\mu = \gamma \cdot \frac{1}{\sum_t \alpha_t \frac{K_t + x_t}{k_t\, x_t} + \beta} = \frac{\gamma}{\sum_t \alpha_t \frac{K_t}{k_t\, x_t} + \sum_t \alpha_t \frac{x_t}{k_t\, x_t} + \beta} = \frac{\gamma}{\sum_t \frac{\alpha''_t}{x_t} + \beta''}, \tag{46}$$

that is, the same form as before, but with parameters $\alpha'' = \frac{\alpha_t K_t}{k_t}$ and $\beta'' = \beta + \sum_t \frac{\alpha_t}{k_t}$. By comparing this to the classical hyperbolic Monod curve

In summary, this means: for a single external metabolite, we obtain a classical hyperbolic Monod curve of the form $\mu = \frac{\mu^{\max} x}{k + K_{\mathrm{Monod}}}$, with the effective parameters $\mu^{\max} = \gamma/\beta$ and $K_{\mathrm{Monod}} = \alpha/\beta$, where primes (in α and β) have been omitted for generality. For Monod functions with several external metabolites, we obtain a similar form; a simple hyperbolic Monod curve with an effective external concentration $x^{\mathrm{eff}} = [\sum_t \frac{\alpha_t}{x_t}]^{-1}$, given by a weighted harmonic mean of the actual external concentrations x_t. For both types of rate laws, the parameters are directly obtained from a single ECM run with the model inquestion, and for a reference state with typical external concentrations.

The function parameters depend on the flux mode assumed, and so far, we assumed that this flux mode was fixed and given (Assumption 1). Now we drop this assumption and assume that the cell can *choose* its optimal flux mode for each external condition (combination of external concentrations). This is quite easy to model: we know that the optimal flux modes are EFMs. For each EFM, we obtain a Monod function of the above form, and to obtain the actual Monod function (assuming an optimal choice of fluxes), we just take the *maximum* among all these functions. With an index j for EFMs, the final Monod function reads:

$$\mu(x_1, .., x_n) = \max_j \frac{\gamma}{\sum_t \frac{\alpha_{tj}}{x_t} + \beta_j}, \tag{47}$$

where primes (in α and β) have again been omitted. Note that, again, this Monod function holds both for linear and for Michaelis-Menten transporter kinetics (which will lead to different choices of the parameter values).

Let us now revisit our above assumptions. Assumtion 1 has already been dropped. As noted before, assumption 2 can also be dropped, leading to a simple nonlinear scaling of our formula. Regarding transporter kinetics, we considered two possible rate laws, and the extension of our formula to other rate laws is straightforward. Thus, we are only left with Assumption 3, the assumption that internal metabolite and enzyme levels are assumed to be constant and that changes in external concentrations are solely compensated by changing transporter levels. This is the main difference between our simple Monod formula and a full calculation by EFCM. How can this assumption be justified? The parameters in our simplified formula are based on a reference state with typical external concentrations and the ensuing optimal enzyme levels. If the external substance concentrations are changing, an adaptation of the transporter level only is always possible, but more costly than an optimal global enzyme adjustment. Therefore, starting from our reference state (and computing the Monod parameters in this state), our Monod function will yield a *lower bound* on the actual Monod function (which is based on a global adjustment). Furtermore, as pointed out in the section on sensitivities, the difference between a local and a distributed, global enzyme adjustent (and thus, the difference between the simplified and the true Monod function) is a second-order effect: in the reference state, the two functions yield the same value (by construction), and close the reference state, the differences will be small. This also means: if we are interested in a specific region of the external concentration space, we can always improve our approximations by choosing a reference state in that region.

5 Model details and elementary flux mode statistics

5.1 Tables with model details

Reaction	EC number	name	KEGG ID	reaction
Glycolysis				
R1	2.7.1.69	pts	RPTSsy	GLU_ext + PEP = GLU_6_P + PYR
R2r	5.3.1.9	pgi	R02740	GLU_6_P = FRU_6_P
R3	2.7.1.11	pfk	R04779	FRU_6_P + ATP = FRU_BIS_P + ADP
R4	3.1.3.11	fbp	R00762	FRU_BIS_P = FRU_6_P
R5r	4.1.2.13	ald	R01070	FRU_BIS_P = DHAP + GA_3P
R6r	5.3.1.1	tim	R01015	GA_3P = DHAP
R7ra	1.2.1.12	gap	R01061	GA_3P + NAD = DPG + NADH
R7rb	2.7.2.3	pgk	R01512	DPG + ADP = PG_3 + ATP
R7rc	5.4.2.11 / 5.4.2.12	pgm	R01518	
R8r	4.2.1.11	pgh	R00658	PG = PEP
R9	2.7.1.40	pyk	R00200	PEP + ADP = PYR + ATP
RR9	2.7.9.2	pps	R00199	PYR + 2 ATP = PEP + 2 ADP
Pentose Phosphate Pathway				
R10a	1.1.1.49	zwf	R00835	GLU_6_P + NAD = GLU_LAC_6_P + NADH
R10b	3.1.1.31	glh	R02035	GLU_LAC_6_P = GLUCO_6_P
R10c	1.1.1.44	pgd	R01528	GLUCO_6_P + NAD = NADH + CO2 + RIBULOSE_5_P
R11r	5.1.3.1	rpe	R01529	RIBULOSE_5_P = XYL_5_P
R12r	5.3.1.6	rpi	R01056	RIBULOSE_5_P = RIBOSE_5_P
R13r	2.2.1.1	txt1	R01641	RIBOSE_5_P + XYL_5_P = SED_7_P + GA_3P
R14r	2.2.1.2	tal	R08575	GA_3P + SED_7_P = ERYTH_4_P + FRU_6_P
R15r	2.2.1.1	txt2	R01067	ERYTH_4_P + XYL_5_P = GA_3P + FRU_6_P
R60	4.2.1.12	edd	R02036	GLUCO_6_P = KDPG
R61r	4.1.2.14	eda	R05605	KDPG = GA_3P + PYR
TCA Cycle				
R20	2.3.1.54	pfl	R00212	PYR + CoASH = ACETYL_CoA + FORMATE
R21	1.2.4.1 & 2.3.1.12	pdh	R00209	PYR + NAD + CoASH = ACETYL_CoA + CO2 + NADH
R22	2.3.3.1	csn	R00351	OXALO + ACETYL_CoA = CITRATE + CoASH
R23r	4.2.1.3	acn	R01324	CITRATE = ISOCIT
R24	1.1.1.41	icd	R00709	ISOCIT + NAD = AKG + NADH + CO2
R25	1.2.4.2	kgd	R08549	AKG + NAD + CoASH = NADH + SUCC_CoA + CO2
R26r	6.2.1.5	scs	R00405	SUCC_CoA + ADP = SUCC + ATP + CoASH
R27	1.3.5.1	sdh	R02164	SUCC + ADP + OXY_ext = FUMARATE + ATP
R27b	1.3.5.4	frd	R02164	FUMARATE + NADH = SUCC + NAD
R28r	4.2.1.2	fum	R01082	FUMARATE = MALATE
R29r	1.1.1.37	mdh	R00342	MALATE + NAD = OXALO + NADH
Anapleurotic Reactions				
R40	4.1.1.31	ppc	R00345	PEP + CO2 = OXALO
R41	1.1.1.38	me	R00214	MALATE + NAD = PYR + NADH + CO2
R42	4.1.1.49	ppck	R00341	OXALO + ATP = PEP + ADP + CO2
Redox-associated reactions				
R53r	1.1.1.27	ldh	R00703	PYR + NADH = LACTATE + NAD
R54ra	1.2.1.10	ada	R00228	ACETYL_CoA + NADH = ACALD + NAD + CoASH
R54rb	1.1.1.1	adh	R00754	ACALD + NADH = ETOH + NAD
R55a	2.3.1.8	pta	R00230	ACETYL_CoA = ACETYL_P + CoASH
R55b	2.7.2.1	ack	R00315	ACETYL_P + ADP = ACETATE + ATP
Biomass Production				
R70		biomass		
Oxidative phosphorylation				
R80		oxphos	ECnadh	NADH + 2 ADP + OXY_ext = NAD + 2 ATP
R82		atpmain		ATP = ADP + ATP_main
Membrane Transport Reactions				
R90		ex_etoh		ETOH = ETOH_ext
R91		ex_ace		ACETATE = ACETATE_ext
R93		ex_nh3		NH3_ext = NH3
R94		ex_lac		LACTATE = LACTATE_ext
R95		ex_suc		SUCC = SUCC_ext
R96		ex_for		FORMATE = FORMATE_ext
R97r		ex_co2		CO2 = CO2_ext

Table S2: Reactions in the model. Reversible reactions are denoted by reaction numbers ending with an r.

Original reaction	New reaction(s)	Reaction formula
R7r	R7ra	GA˙3P + NAD = DPG + NADH
	R7rb	DPG + ADP = PG3 + ATP
	R7rc	PG3 = PG
R10	R10a	GLU˙6˙P + NAD = GLULAC6P + NADH
	R10b	GLULAC6P = GLUCO6P
	R10c	GLUCO6P + NAD = NADH + CO2 + RUBILOSE˙5˙P
R27r	R27	SUCC + ADP + OXYext = FUMURATE + ATP
R81		merged with R27
R27r	R27b	FUMERATE + NADH = SUCC + NAD
R83		merged with R27b
R54r	R54ra	ACETYL˙CoA + NADH = ACALD + NAD + CoASH
	R54rb	ACALD + NADH = ETOH + NAD
R55r	R55a	ACETYL˙CoA + ADP = ACETYLP + CoASH
	R55b	ACETYLP + ADP = ACETATE + ATP
new	R60	GLUCO6P = KDGP
new	R61r	KDGP = GA3P + PYR

Table S3: Changes in reactions compared to the original model in Carlson et al. [20].

Name	KEGG ID	Stoichiometry	Molecular mass [mg mmol^{-1}]	Weight in biomass [mg mmol^{-1}]	#Carbons	#Carbons in biomass
acetyl-CoA	C00024	-41	59	-2419	2	-82
α-ketoglutarate	C00026	-14	146	-2044	5	-70
CO2	C00011	2	44	88	1	2
DHAP	C00236	-5	266	-1330	3	-15
glucose-6P	C00345	-4	180	-720	6	-24
NH_3	C00014	-139	17	-2363	0	0
oxaloacetate	C00007	-24	132	-3168	4	-96
PEP	C00631	-32	88	-2816	3	-96
pyruvate	C00022	-38	88	-3344	3	-11
ribose-5P	C00117	-13	150	-1950	5	-65
erythrose-4P	C00279	-5	120	-600	4	-20
TOTAL				**-20666**		**-580**

Table S4: Description of the biomass-producing reaction

Reaction	k_{cat} value	Reaction	k_{cat} value	Reaction	k_{cat} value
R1	103.9	R13r	46.4	R53r	139.4
R2r	321.9	R14r	16.7	R54ra	0.35
R3	115.0	R15r	75.3	R54rb	324.1
R4	25.4	R20	4807.8	R55a	91.3
R5r	8.0	R21	37.7	R55b	58.8
R6r	7838.0	R22	358.7	R60	247.5
R7ra	233.2	R23r	32.6	R61r	79.9
R7rb	390.2	R24	108.5	R70	99.5
R7rc	53.1	R25	150.6	R80	4.00E+06
R8r	210.9	R26r	89.2	R82	180.6
R9	513.1	R27	78.1	R90	100.2
RR9	13.2	R27b	179.3	R91	100.1
R10a	241.0	R28r	281.7	R93	99.7
R10b	409.8	R29r	210.7	R94	100.1
R10c	110.2	R40	123.2	R95	100.0
R11r	133.9	R41	75.9	R96	100.1
R12r	1362.0	R42	51.5	R97r	100.1

Table S5: k_{cat} values used in the model (in s^{-1})

Reaction	Metabolite	K_M value	Reaction	Metabolite	K_M value	Reaction	Metabolite	K_M value
R1	glu6p	0.101735	R15r	fru6p	0.736973	R42	oxalo	0.570976
R1	glucoseExt	0.11626	R15r	ga3p	1.269744	R42	pep	0.064276
R1	pep	0.098295	R15r	xyl5p	0.151503	R53r	lactate	0.517374
R1	pyr	0.101735	R20	acetylcoa	0.035199	R53r	nad	0.517374
R2r	fru6p	0.162398	R20	coash	0.016796	R53r	nadh	0.019328
R2r	glu6p	0.272797	R20	formate	6.347527	R53r	pyr	0.019328
R3	adp	0.113417	R20	pyr	2.180238	R54ra	acetylcoa	0.024179
R3	atp	0.140957	R21	acetylcoa	0.158941	R54ra	acald	1.801732
R3	fru6p	0.116272	R21	co2	0.158941	R54ra	coash	0.00786
R3	frubisp	0.113417	R21	coash	0.062916	R54ra	nad	0.041481
R4	fru6p	0.170733	R21	nad	0.062916	R54ra	nadh	0.112985
R4	frubisp	0.016115	R21	nadh	0.158941	R54rb	acald	0.059298
R5r	dhap	0.078151	R21	pyr	0.29068	R54rb	etoh	5.494644
R5r	frubisp	0.204365	R22	acetylcoa	0.08665	R54rb	nad	0.168638
R5r	ga3p	0.078151	R22	citrate	0.075572	R54rb	nadh	0.059298
R6r	dhap	0.074992	R22	coash	0.075572	R55a	acetylcoa	0.042439
R6r	ga3p	0.744648	R22	oxalo	0.028686	R55a	acetylp	0.312643
R7ra	dpg	0.05757	R23r	citrate	3.48647	R55a	coash	0.085985
R7ra	ga3p	0.686739	R23r	isocit	2.420319	R55b	acetate	3.435988
R7ra	nad	0.055756	R24	akg	0.48257	R55b	acetylp	0.154167
R7ra	nadh	0.05757	R24	co2	2.022353	R55b	adp	0.402085
R7rb	adp	0.042553	R24	isocit	0.022669	R55b	atp	0.071354
R7rb	atp	0.235002	R24	nad	1.064576	R60	gluco6p	0.043396
R7rb	dpg	0.042553	R24	nadh	0.011912	R60	kdpg	0.149596
R7rb	pg3	0.235002	R25	akg	0.067023	R61r	ga3p	0.001465
R7rc	pg3	0.132459	R25	co2	0.107909	R61r	kdpg	0.560779
R7rc	pg	0.075495	R25	coash	0.092671	R61r	pyr	0.001465
R8r	pep	0.130673	R25	nad	0.092671	R70	acetylcoa	0.61
R8r	pg	0.108362	R25	nadh	0.107909	R70	akg	0.44
R9	adp	0.218366	R25	succcoa	0.107909	R70	coash	1.37
R9	atp	8.453199	R26r	adp	0.056003	R70	eryth4p	0.03
R9	pep	0.2914	R26r	atp	0.081214	R70	glu6p	8.75
R9	pyr	0.047641	R26r	coash	0.007309	R70	nad	2.55
RR9	adp	0.087324	R26r	succ	0.237	R70	nadh	0.08
RR9	atp	0.035013	R26r	succcoa	0.010492	R70	nh3	0.01
RR9	pep	0.093447	R27	adp	0.370552	R70	oxalo	0.00
RR9	pyr	0.086415	R27	atp	0.026987	R70	pep	0.18
R10a	glu6p	0.313906	R27	fumarate	0.081233	R70	pyr	0.39
R10a	glulac6p	0.128734	R27	oxygen	0.370552	R70	ribose5p	1.32
R10a	nad	0.863228	R27	succ	0.075581	R80	adp	0.001
R10a	nadh	0.128734	R27b	fumarate	0.020135	R80	atp	1000
R10b	glulac6p	0.168255	R27b	nad	0.043142	R80	nad	1000
R10b	gluco6p	0.059434	R27b	nadh	0.231791	R80	nadh	0.001
R10c	co2	0.062615	R27b	succ	0.205437	R80	oxygen	1000
R10c	gluco6p	0.101332	R28r	fumarate	0.313894	R82	adp	0.13004
R10c	nad	0.059104	R28r	malate	0.61515	R82	atp	0.076899
R10c	nadh	0.062615	R29r	malate	3.189696	R82	ATPmain	0.13004
R10c	ribulose5p	0.062615	R29r	nad	0.459655	R90	etoh	0.099929
R11r	ribulose5p	0.087822	R29r	nadh	0.032069	R90	ethanolExt	0.100071
R11r	xyl5p	0.113867	R29r	oxalo	0.028266	R91	acetate	0.099946
R12r	ribose5p	1.247289	R40	co2	0.114505	R91	acetateExt	0.100054
R12r	ribulose5p	0.558142	R40	oxalo	0.04259	R93	nh3	0.099855
R13r	ga3p	1.22708	R40	pep	0.363706	R93	nh3Ext	0.100145
R13r	ribose5p	0.97228	R41	co2	0.088485	R94	lactate	0.099963
R13r	sed7p	2.110123	R41	malate	0.361402	R94	lactateExt	0.100037
R13r	xyl5p	0.15677	R41	nad	0.069121	R95	succ	0.099985
R14r	eryth4p	0.175159	R41	nadh	0.088485	R95	succinateExt	0.100015
R14r	fru6p	0.888093	R41	pyr	0.088485	R96	formate	0.099939
R14r	ga3p	0.577958	R42	adp	0.04843	R96	formateExt	0.100061
R14r	sed7p	0.206421	R42	atp	0.074986	R97r	co2	0.099947
R15r	eryth4p	0.093367	R42	co2	5.210404	R97r	co2Ext	0.100053

Table S6: K_M values used in the model (in mM)

Reaction	K_{eq} value	Reaction	k_{cat} value
R2r	0.50987024	R14r	0.89996461
R5r	0.000297615	R15r	37.50713
R6r	11.249283	R23r	0.073843788
R7ra	0.088486876	R26r	0.52174352
R7rb	727.33174	R28r	4.7316568
R7rc	0.16087119	R29r	6.11036E-05
R8r	3.5014543	R53r	20577.22
R11r	2.3258292	R54ra	0.002258241
R12r	2.3233211	R54rb	2767.6607
R13r	3.6538527	R61r	0.009573533

Table S7: K_{eq} values used in the model

Reaction	Molecular mass	Reaction	Molecular mass	Reaction	Molecular mass
R1	255848	R13r	73043	R53r	36535
R2r	61530	R14r	35219	R54ra	96127
R3	139360	R15r	73043	R54rb	96127
R4	36834	R20	85357	R55a	77172
R5r	39147	R21	282880	R55b	43290
R6r	53940	R22	96020	R60	64600
R7ra	35530	R23r	95587.5	R61r	22300
R7rb	41120	R24	45760	R70	59821.5
R7rc	28556	R25	1.21E+06	R80	910000
R8r	45650	R26r	71170	R82	59821.5
R9	50000	R27	792299	R91	59197
RR9	87430	R27b	181038.5	R93	44515
R10a	55700	R28r	60000	R94	59168
R10b	36310	R29r	32337	R95	45436
R10c	102960	R40	198120	R96	30991
R11r	24554	R41	63197		
R12r	19466.5	R42	59643		

Table S8: Enzyme molecule masses used in the model (in Daltons)

Compounds			fixed total concentration (mM)
atp	adp		10.185
nad	nadh		2.6332
coash	succoa	acetylcoa	11.249283

Table S9: Cofactor pairs. In the model, some cofactors are not newly synthesized by reactions, but only interconverted in pairs. We have fixed the total concentration for these metabolites.

5.2 Sampling of flux modes near the Pareto front

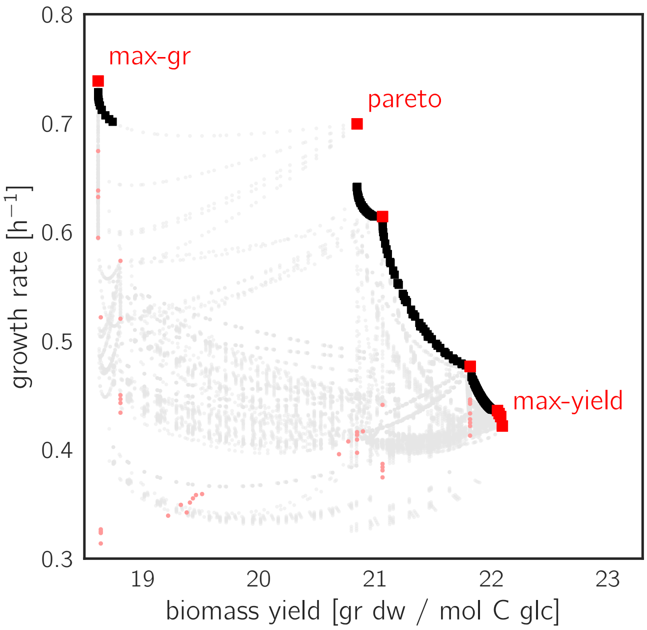

Figure S24: **Flux modes and EFMs near the Pareto front.** To determine the shape of the Pareto front (containing any flux modes, not only EFMs), we first considered EFMs that are either Pareto-optimal EFMs or among the highest-ranking EFMs in terms of yield or growth rate. Then we sampled flux modes that are convex combinations of a few of these EFMs. Grey dots represent the samples, light red dots are EFMs, large red squares are Pareto-optimal EFMs and black squares are those sampled non-elementary flows that turned out to be Pareto-optimal. Pareto-optimal EFMs must also be Pareto-optimal among EFMs, i.e. they must not be dominated by any other EFM; however, the converse need not hold in all cases: there can be EFMs that are Pareto-optimal among EFMs, but not among all flux modes. Note that the figure is zoomed to show the entire span of the Pareto front in detail.

5.3 Selected elementary flux modes

Acronym*	EFM #	Biomass yield [g/C-mol]	Growth rate [h^{-1}]†	Oxygen uptake	Acetate secre-tion	Lactate secre-tion	Succinate secre-tion	fraction PPP	# active reactions
max-gr	1565	18.6	0.739	0.49	0	0	0	2.77	24
pareto	1218	20.8	0.699	0.42	0	0	0	2.50	25
max-yield	938	22.1	0.422	0.39	0	0	0	0	28
ana-lac	1295	2.1	0.258	0	0	0.92	0.02	0.90	31
aero-ace	559	15.8	0.520	0.21	0.35	0	0	0.11	28
exp	9999	17.7	0.409	0.29	0.22	0	0	0.39	38

*max-gr: maximum growth rate; max-yield: maximum yield; ana-lac: anaerobic lactate fermentation; aero-suc: aerobic succinate fermentation; aero-ace: aerobic acetate fermentation; exp: experimentally measured flux distribution †Growth rate is given for standard conditions, where [glucose] = 100 mM, and [O_2] = 0.21 mM.

Table S10: Details on selected EFMs representing different growth strategies. Metabolic fluxes are given in carbon moles (or O_2 moles) per carbon mole of glucose taken up. The flux fraction through the pentose phosphate pathway (PPP) is defined as the ratio R1/R10a (see Figure S4 for the reaction numbers in the network).

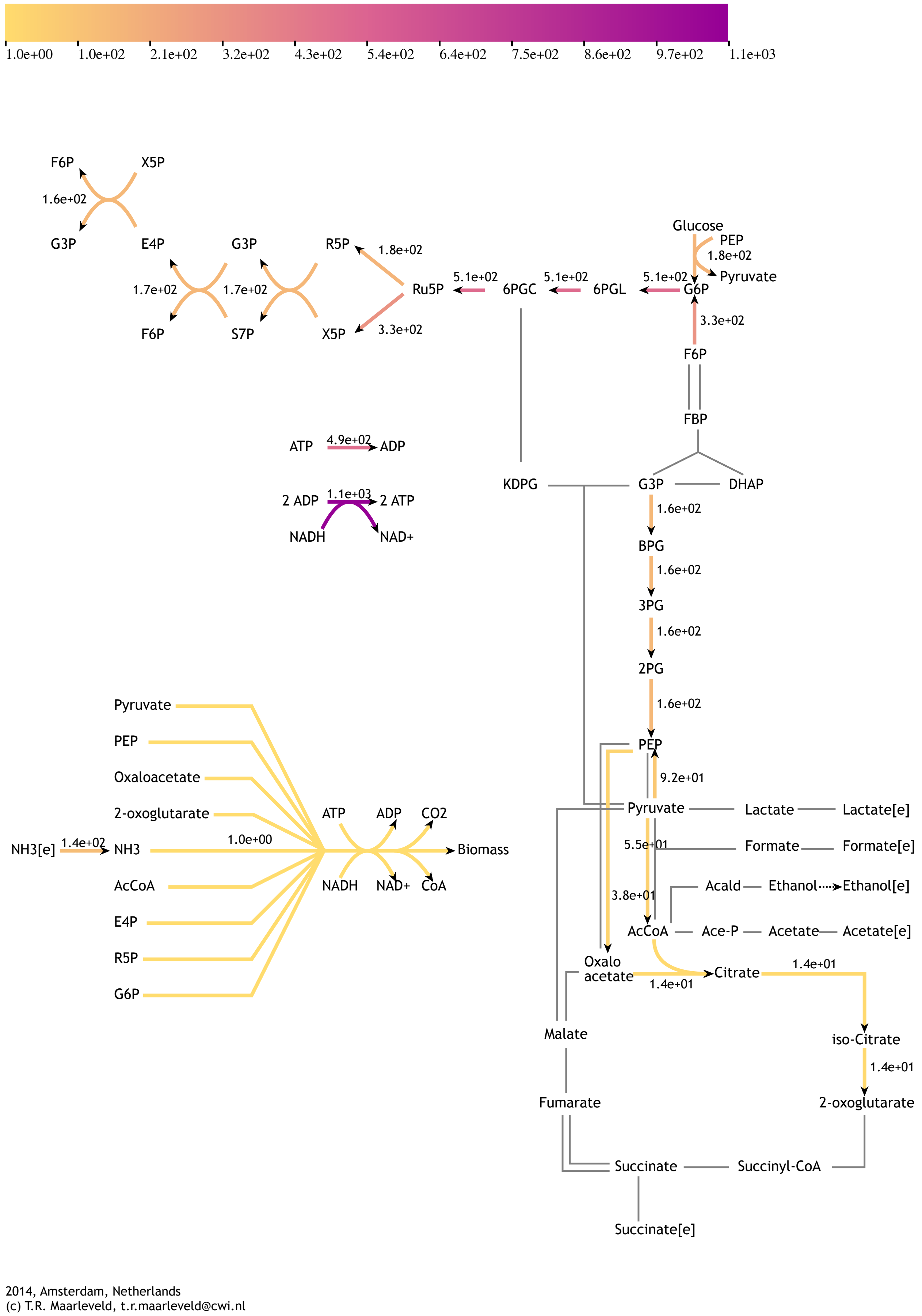

Figure S25: **The flux distribution of *max-gr* (EFM #1565).**

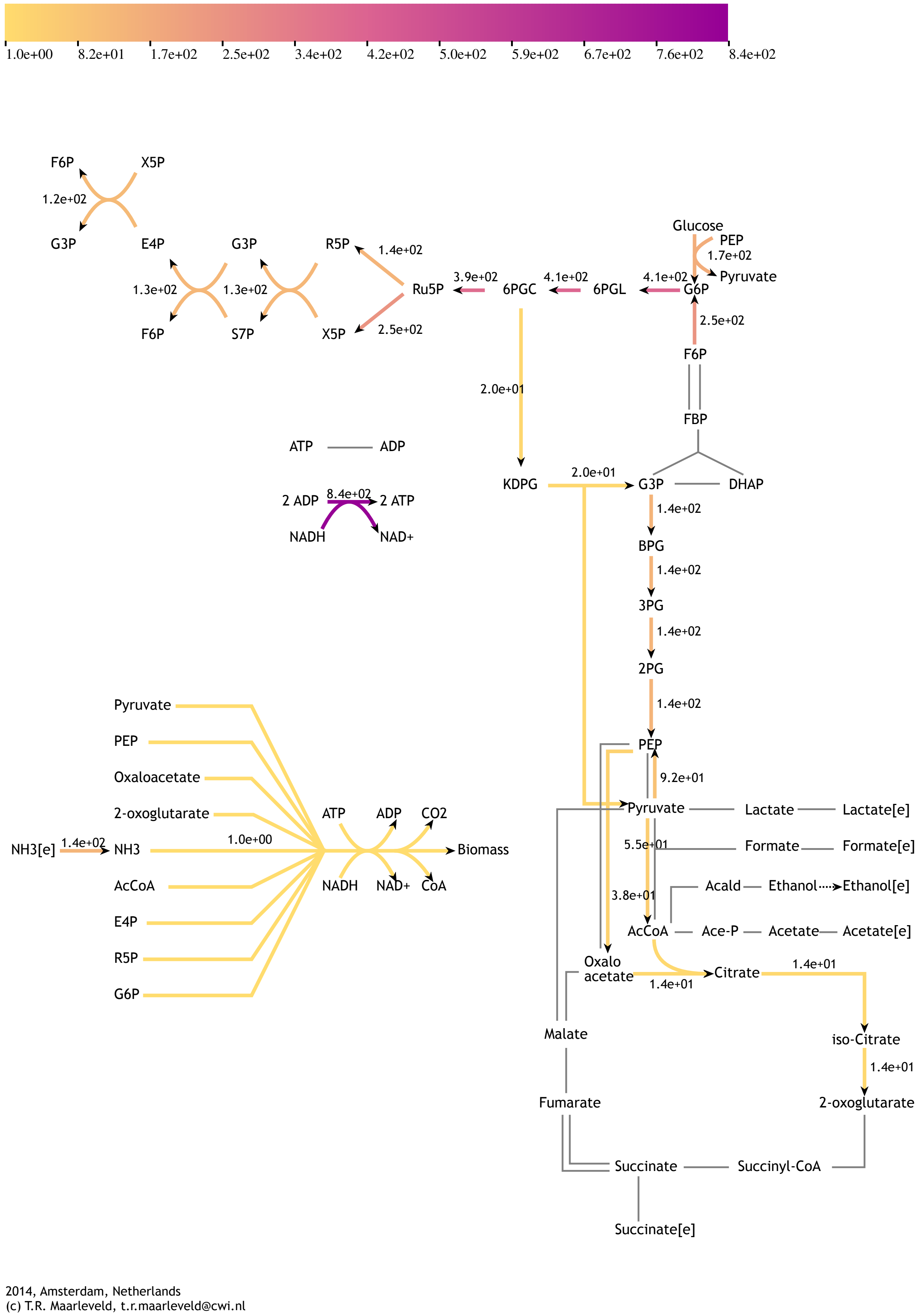

Figure S26: **The flux distribution of *pareto* (EFM #1218).**

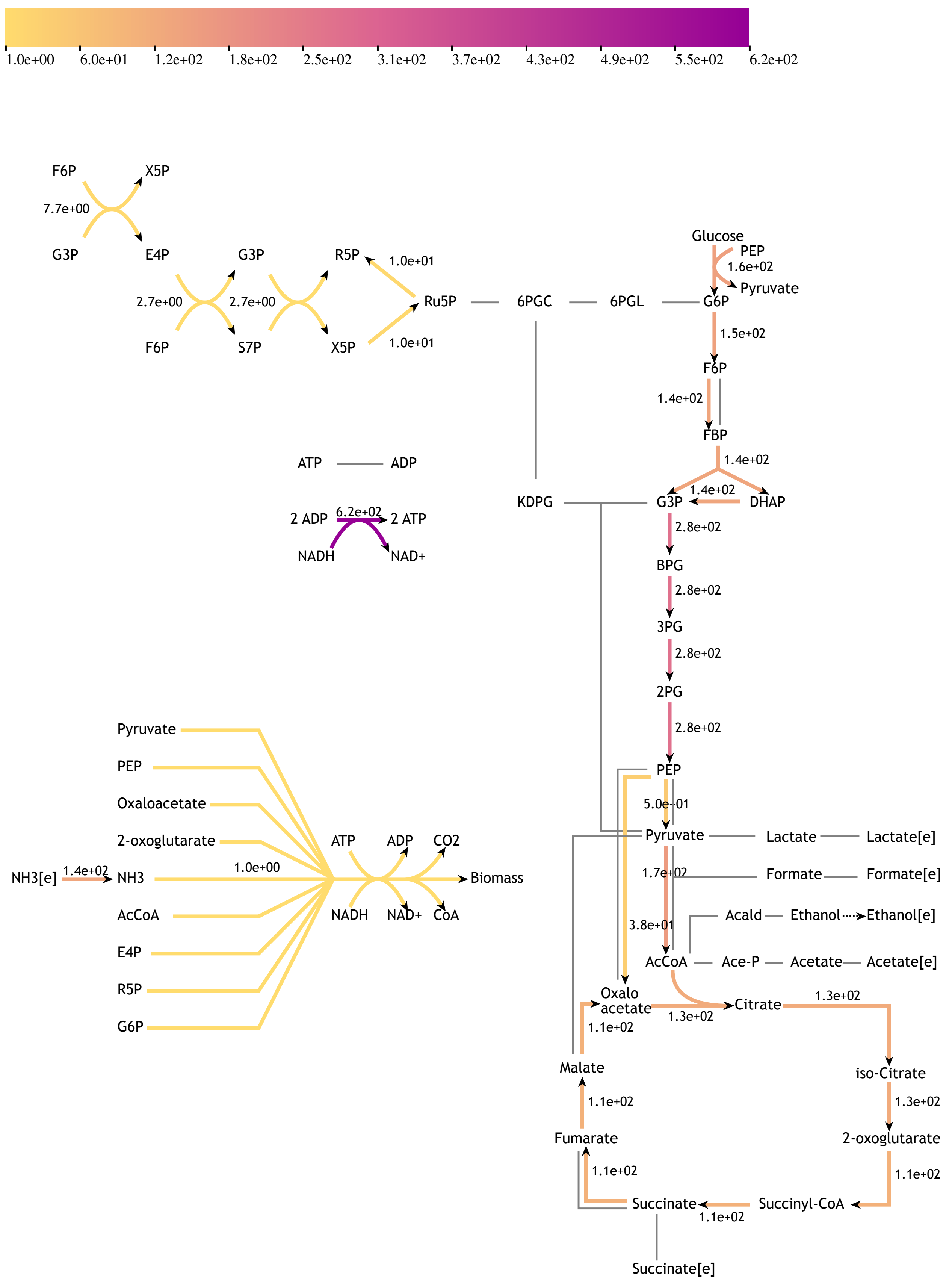

2014, Amsterdam, Netherlands
(c) T.R. Maarleveld, t.r.maarleveld@cwi.nl

Figure S27: **The flux distribution of *max-yield* (EFM #938).**

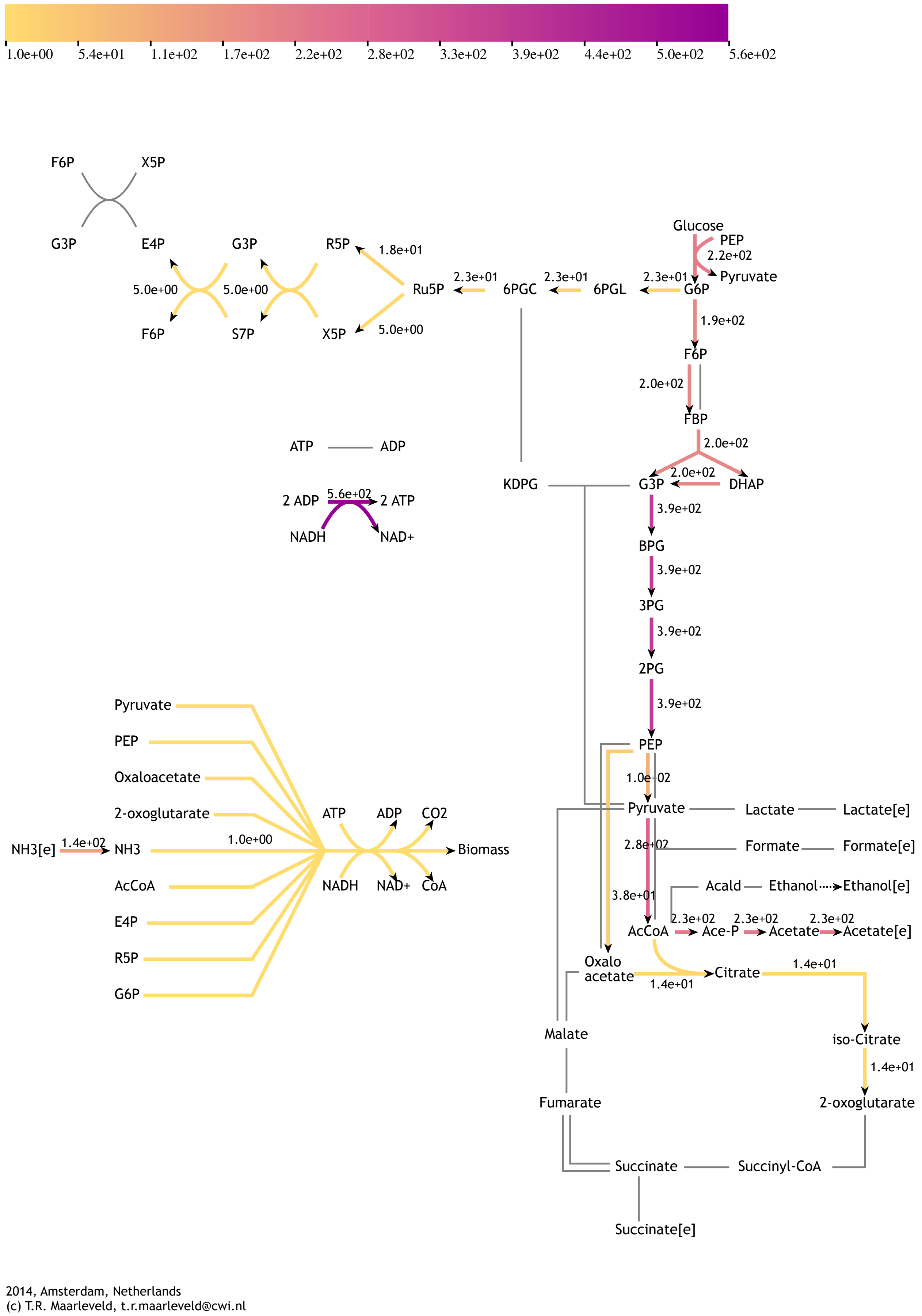

Figure S28: **The flux distribution of *aero-ace* (EFM #1155).**

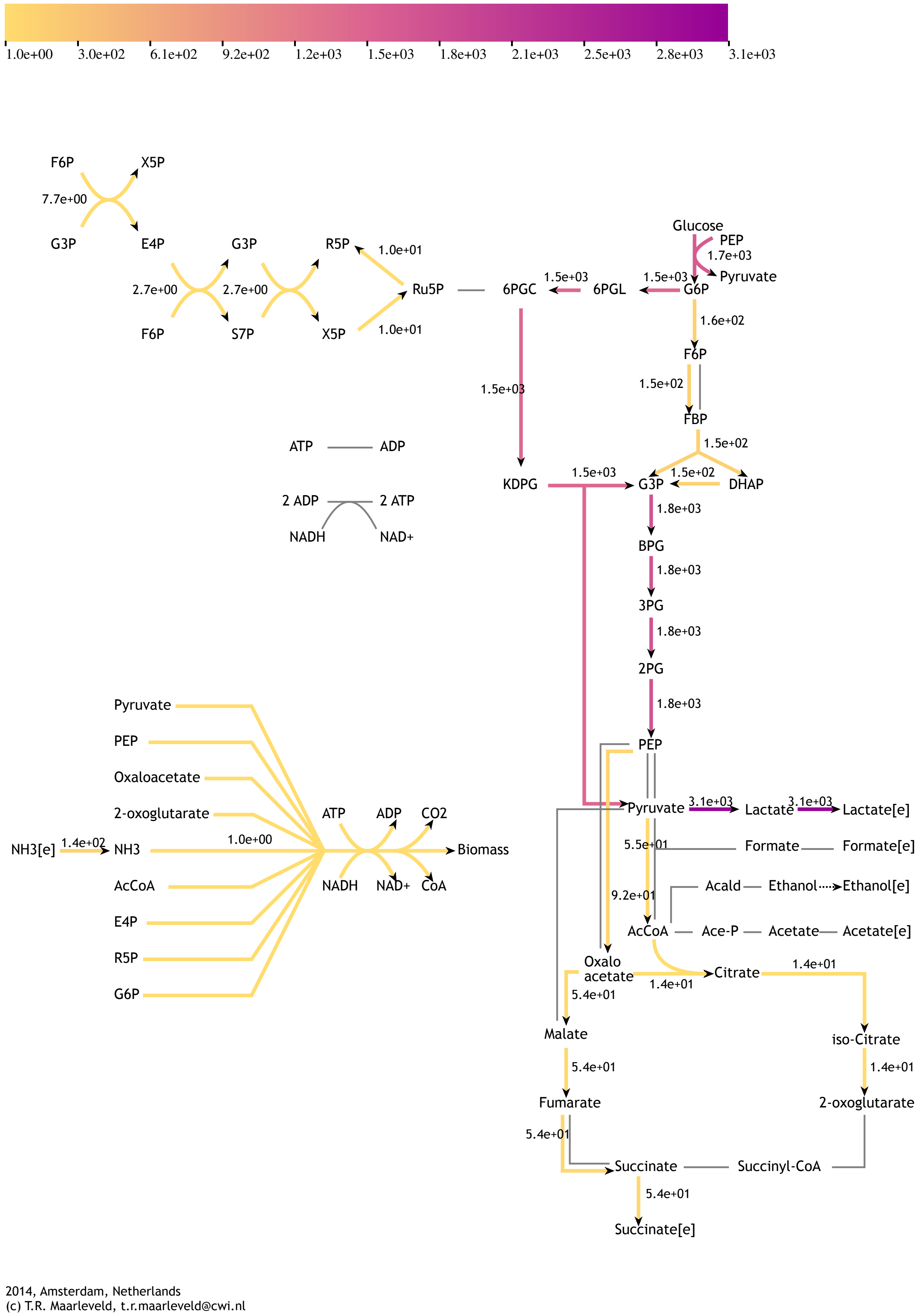

Figure S29: **The flux distribution of *ana-lac* (EFM #1295).**

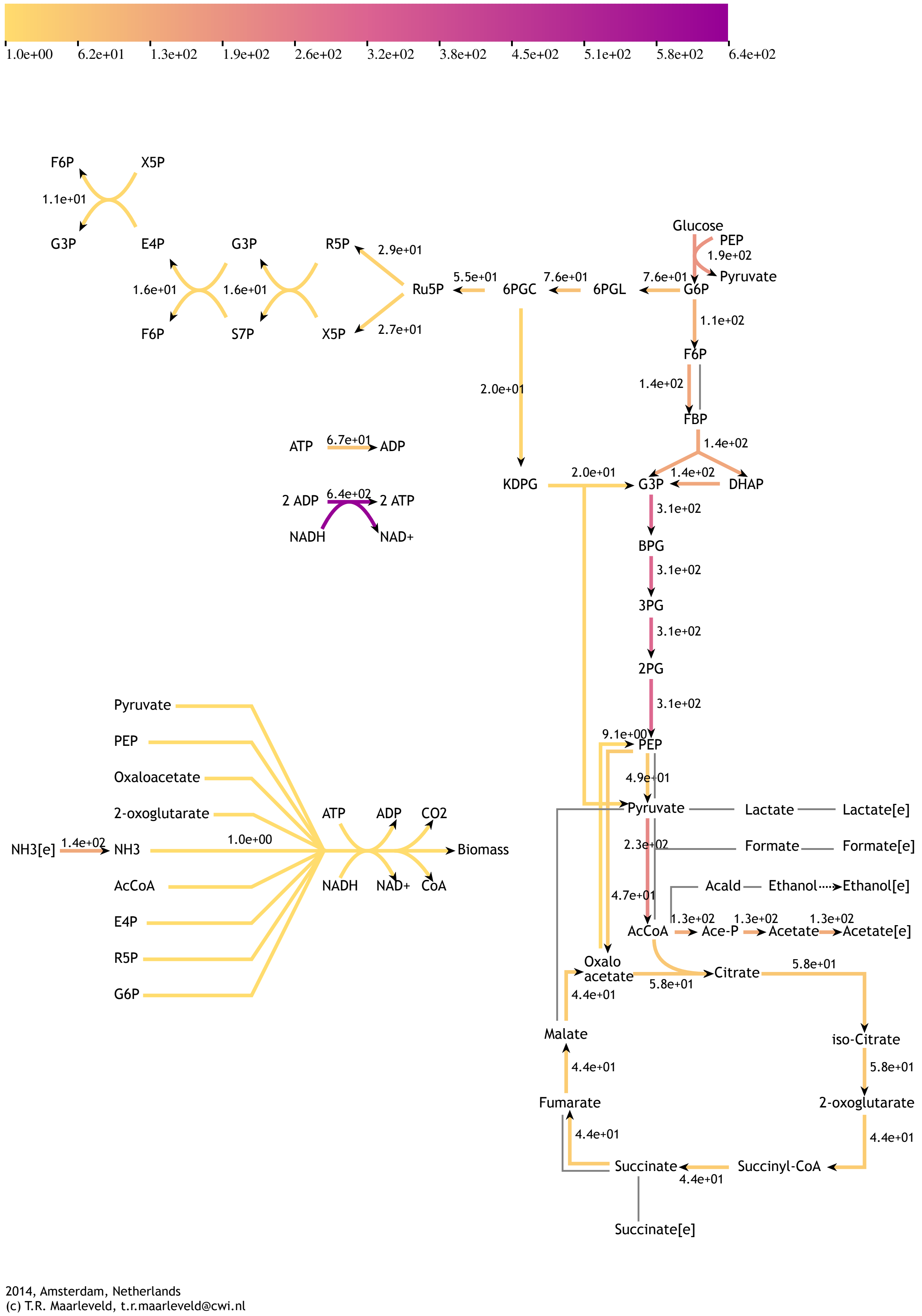

Figure S30: **The flux distribution of *exp*.**

6 List of supplementary data files

Data files describing the *E. coli model* and the analysis of metabolic strategies therein. The files can be downloaded from github: `https://github.com/liebermeister/enzyme-flux-cost-minimization`.

- `Ecoli_Central_Metabolism.xlsx` – reactions, metabolites, stoichiometry matrix and EFMs
- `Ecoli_Central_Metabolism_SBML.xml` – SBML file of the central metabolism network of *E. coli*
- `Kinetic_Constants_Original.xlsx` – kinetic parameters obtained from literature with references
- `Kinetic_Constants_Original.tsv` – (averages of the) literature data; input for parameter balancing
- `Kinetic_Constants_Balanced.tsv` – balanced parameter values (used in the model)
- `Ecoli_Model_Data_NEOS.zip` – multi-file NEOS input for optimization of enzyme levels
- `Ecoli_Model_Data_NEOS_SBtab.csv` – single-file (SBtab) NEOS input for enzyme level optimization
- `GAMS_run_files.zip` – GAMS code files
- `Sensitivities_Kcat.csv` – cost and growth sensitivities of the k_{cat} values under standard conditions
- `Sensitivities_Keq.csv` – cost and growth sensitivities of the k_{eq} values under standard conditions
- `Sensitivities_KM.csv` – cost and growth sensitivities of the K_M values under standard conditions
- `Anaerobic_EFM_Results.csv` – results for relevant EFMs in anaerobic conditions
- `Anaerobic_Enzyme_Abundance.csv` – individual enzyme abundances for all relevant EFMs in anaerobic conditions
- `Aerobic_EFM_Results.csv` – results for relevant EFMs in standard aerobic conditions
- `Aerobic_Enzyme_Abundance.csv` – individual enzyme abundances for all relevant EFMs in standard aerobic conditions

References

[1] T. Lubitz, M. Schulz, E. Klipp, and W. Liebermeister. Parameter balancing for kinetic models of cell metabolism. *Journal of Physical Chemistry B*, 114(49):16298–16303, 2010.

[2] A. Brooke, D. Kendrick, A. Meeraus, and R. Raman. The general algebraic modeling system. *GAMS Development Corporation*, 1998.

[3] W. Liebermeister, J. Uhlendorf, and E. Klipp. Modular rate laws for enzymatic reactions: Thermodynamics, elasticities, and implementation. *Bioinformatics*, 26(12):1528–1534, 2010.

[4] R. Milo, P. Jorgensen, U. Moran, G. Weber, and M. Springer. Bionumbers – the database of key numbers in molecular and cell biology. *Nucleic Acids Research*, 38(Database issue):D750–D753, 2010.

[5] K. Valgepea, K. Adamberg, A. Seiman, and R. Vilu. Escherichia coli achieves faster growth by increasing catalytic and translation rates of proteins. *Molecular Biosystems*, 9(9):2344–23458, 2013.

[6] M. Scott, C.W. Gunderson, E.M. Mateescu, Z. Zhang, and T. Hwa. Interdependence of cell growth and gene expression: Origins and consequences. *Science*, 330:1099, 2010.

[7] E. Noor, A. Flamholz, A. Bar-Even, D. Davidi, R. Milo, and W. Liebermeister. The protein cost of metabolic fluxes: Prediction from enzymatic rate laws and cost minimization. *PLoS Computational Biology*, 12(10):e1005167, 2016.

[8] H.-G. Holzhütter. The principle of flux minimization and its application to estimate stationary fluxes in metabolic networks. *European Journal of Biochemistry*, 271(14):2905–2922, 2004.

[9] Q. K. Beg, A. Vazquez, J. Ernst, M.A. de Menezes, Z. Bar-Joseph, A.L. Barabási, and Z.N. Oltvai. Intracellular crowding defines the mode and sequence of substrate uptake by Escherichia coli and constrains its metabolic activity. *Proceedings of the National Academy of Sciences*, 104(31):12663–12668, 2007.

[10] M. Mori, T. Hwa, O.C. Martin, A. De Martino, and E. Marinari. Constrained allocation flux balance analysis. *PLoS Computational. Biology*, 12(6):e1004913, 2016.

[11] E.J. O'Brien, J. Utrilla, and B.O. Palsson. Quantification and classification of E. coli proteome utilization and unused protein costs across environments. *PLoS Computational Biology*, 12(6):e1004998, 2016.

[12] S. Müller, G. Regensburger, and R. Steuer. Resource allocation in metabolic networks: Kinetic optimization and approximations by FBA. *Biochemical Society Transactions*, 43:1195–1200, 2015.

[13] M. Mori, T. Hwa, O.C. Martin, A. De Martino, and E. Marinari. Constrained allocation flux balance analysis. *PLoS Computational Biology*, 12(6):e1004913, 2016.

[14] A. Goelzer, V. Fromion, and G. Scorletti. Cell design in bacteria as a convex optimization problem. *Automatica*, 47(6):1210–1218, 2011.

[15] A. Goelzer and V. Fromion. Bacterial growth rate reflects a bottleneck in resource allocation. *Biochimica et Biophysica Acta (BBA) - General Subjects*, 1810(10):978–988, 2011.

[16] S. Tabe-Bordbar and S.A. Marashi. Finding elementary flux modes in metabolic networks based on flux balance analysis and flux coupling analysis: Application to the analysis of Escherichia coli metabolism. *Biotechnology Letters*, 35(12):2039–44, 2013.

[17] S. Schuster, T. Pfeiffer, F. Moldenhauer, I. Koch, and T. Dandekar. Exploring the pathway structure of metabolism: Decomposition into subnetworks and application to Mycoplasma pneumoniae. *Bioinformatics*, 18(2):351–361, 2002.

[18] T.R. Maarleveld, M.T. Wortel, B.G. Olivier, B. Teusink, and F.J. Bruggeman. Interplay between constraints, objectives, and optimality for genome-scale stoichiometric models. *PLoS Computational Biology*, 11(4):e1004166, 2015.

[19] R.P. Carlson. Metabolic systems cost-benefit analysis for interpreting network structure and regulation. *Bioinformatics*, 23(10):1258–1264, 2007.

[20] R. Carlson and F. Srienc. Fundamental Escherichia coli biochemical pathways for biomass and energy production: Identification of reactions. *Biotechnology and Bioengineering*, 85(1):1–19, 2004.

[21] M. Terzer and J. Stelling. Large-scale computation of elementary flux modes with bit pattern trees. *Bioinformatics*, 24(19):2229–2235, 2008.

[22] Y.-F. Xu, D. Amador-Noguez, M.L. Reaves, X.-J. Feng, and J.D. Rabinowitz. Ultrasensitive regulation of anaplerosis via allosteric activation of PEP carboxylase. *Nature Chemical Biology*, 8(6):562–568, 2012.

[23] J.S. Hofmeyr, O.P.C. Gqwaka, and J.M. Rohwer. A generic rate equation for catalysed, template-directed polymerisation. *FEBS Letters*, 587:2868–2875, 2013.

[24] A. Bar-Even, E. Noor, Y. Savir, W. Liebermeister, D. Davidi, D.S. Tawfik, and R. Milo. The moderately efficient enzyme: Evolutionary and physicochemical trends shaping enzyme parameters. *Biochemistry*, 50(21):4402–4410, 2011.

[25] L. R. Bakken and R. A. Olsen. Buoyant densities and dry-matter contents of microorganisms: Conversion of a measured biovolume into biomass. *Applied and Environmental Microbiology*, 45(4):1188–1195, 1983.

[26] P. Millard, K. Smallbone, and P. Mendes. Metabolic regulation is sufficient for global and robust coordination of glucose uptake, catabolism, energy production and growth in Escherichia coli. *PLOS Computational Biology*, 13(2):e1005396, 2017.

[27] L.J.P. van der Maaten and G.E. Hinton. Visualizing high-dimensional data using t-SNE. *Journal of Machine Learning Research*, 9:2579–2605, 2008.

[28] L. Gerosa, B.R.B. Haverkorn van Rijsewijk, D. Christodoulou, K. Kochanowski, T.S.B. Schmidt, E. Noor, and U. Sauer. Pseudo-transition analysis identifies the key regulators of dynamic metabolic adaptations from steady-state data. *Cell Systems*, 1(1):1, 2015.

[29] A. Schmidt, Kochanowski K, S. Vedelaar, E. Ahrné, B. Volkmer, L. Callipo, K. Knoops, M. Bauer, R. Aebersold, and M. Heinemann. The quantitative and condition-dependent Escherichia coli proteome. *Nature Biotechnology*, 34(1):104–110, 2016.

[30] A. Flamholz, E. Noor, A. Bar-Even, W. Liebermeister, and R. Milo. Glycolytic strategy as a tradeoff between energy yield and protein cost. *Proceedings of the National Academy of Sciences*, 110(24): 10039–10044, 2013.

[31] D. Segrè, A. DeLuna, M.G. Church, and R. Kishony. Modular epistasis in yeast metabolism. *Nature Genetics*, 37:77–83, 2005.
